# Sunlight-driven nitrate loss records Antarctic surface mass balance

**DOI:** 10.1038/s41467-022-31855-7

**Published:** 2022-07-25

**Authors:** Pete D. Akers, Joël Savarino, Nicolas Caillon, Aymeric P. M. Servettaz, Emmanuel Le Meur, Olivier Magand, Jean Martins, Cécile Agosta, Peter Crockford, Kanon Kobayashi, Shohei Hattori, Mark Curran, Tas van Ommen, Lenneke Jong, Jason L. Roberts

**Affiliations:** 1 Université Grenoble Alpes, CNRS, IRD, Grenoble INP, IGE, Grenoble, France; 2 Department of Geography, Trinity College Dublin, Dublin, Ireland; 3 Japan Agency for Marine-Earth Science and Technology, Yokosuka, Japan; 4 Laboratoire des Sciences du Climat et de l’Environnement, LSCE-IPSL, CEA-CNRS-UVSQ, Université Paris-Saclay, Gif-sur-Yvette, France; 5 Department of Marine Chemistry and Geochemistry, Woods Hole Oceanographic Institution, Woods Hole, MA USA; 6 Department of Earth and Planetary Sciences, Harvard University, Cambridge, MA USA; 7 Department of Chemical Science and Engineering, Tokyo Institute of Technology, Yokohama, Japan; 8 International Center for Isotope Effects Research, Nanjing University, Nanjing, China; 9 School of Earth Sciences and Engineering, Nanjing University, Nanjing, China; 10 Australian Antarctic Division, Department of Climate Change, Energy, the Environment and Water, Kingston, TAS Australia; 11 Australian Antarctic Program Partnership, Institute of Marine and Antarctic Studies, University of Tasmania, Hobart, TAS Australia

**Keywords:** Environmental chemistry, Cryospheric science, Palaeoclimate

## Abstract

Standard proxies for reconstructing surface mass balance (SMB) in Antarctic ice cores are often inaccurate or coarsely resolved when applied to more complicated environments away from dome summits. Here, we propose an alternative SMB proxy based on photolytic fractionation of nitrogen isotopes in nitrate observed at 114 sites throughout East Antarctica. Applying this proxy approach to nitrate in a shallow core drilled at a moderate SMB site (Aurora Basin North), we reconstruct 700 years of SMB changes that agree well with changes estimated from ice core density and upstream surface topography. For the under-sampled transition zones between dome summits and the coast, we show that this proxy can provide past and present SMB values that reflect the immediate local environment and are derived independently from existing techniques.

## Abstract (web summary)

Snow accumulation rates in Antarctica can now be reconstructed from nitrate isotopes in snow and ice. This independent technique offers scientists a new tool for studying how Antarctic climate changed in the past and how it may change in the future.

## Introduction

Antarctica holds a critical role in the Earth’s hydrosphere, providing long-term storage of 27 million km^3^ of ice^1^ and impacting global ocean and atmosphere circulation through its albedo, topography, export of calved glacial ice, and function as an atmospheric heat sink^2–5^. Since even small shifts in the surface mass balance (SMB) across Antarctic ice sheets can redistribute huge masses of water between the cryosphere, ocean, and atmosphere, a clear understanding of how its SMB has responded to past climate change is crucial for calibrating forecast models of the global environment and properly interpreting ice cores^6–10^. Despite this pressing importance, much of Antarctica has insufficient records of both modern and past SMB values, particularly in the transitional zone between the <1000 m elevation wet coastal periphery and the >3000 m elevation ultra-dry dome summits. Although this transitional zone comprises 50% of Antarctica’s surface area^11^, it hosts few long-term scientific stations and is much less targeted for intensive scientific research and deep (>100 m) ice core studies. Because this zone has a highly dynamic SMB system affected by strong wind-driven transport, rugged small scale surface features, and infrequent but high impact precipitation events, our lack of dedicated studies of the transitional zone impedes a comprehensive understanding of past and present SMB changes in Antarctica.

This lack of data is largely a result of logistical challenges with observing the intermediate SMB values in this transitional zone when using existing techniques. Determining modern SMB for new sites typically requires either installing stake transects that need multiple return visits spanning several years or coring several meters of firn to identify the increasingly buried 1992 Pinatubo volcanic horizon with geochemical analysis. However, the limited time and resources during research expeditions to remote areas usually prevents intensive modern SMB surveys with these methods, and, as a result, existing SMB records in the transition zone are largely restricted to a few frequently traveled supply traverse routes^12^. This has left vast regions of Antarctica with no ground-verified SMB data.

Although ice cores have been drilled from a few sites in the transitional zone, extracting SMB histories from these cores is often difficult. At interior dome sites, proxy air temperature from water isotopes (*δ*^2^H or *δ*^18^O) is used to derive snow accumulation rate through water vapor saturation^10^. However, this approach does not account for wind-driven transport and sublimation of surface snow at warmer and lower elevation sites^13–15^. Additionally, water isotopes reflect many environmental factors other than temperature, such as atmospheric circulation changes, transport pathways, and moisture sources, which can lead to large uncertainty and/or bias in reconstructed SMB^16,17^. Changes in ice density along the cores may be converted to SMB provided that they are well-dated, but density-based reconstructions become increasingly uncertain with depth due to thinning and deformation of ice layers^18^ and may be impossible in zones with heavy ice deformation. Cores are also commonly damaged during the drilling and transportation process, and this can make accurate physical measurements of mass and volume very difficult, particularly for the shallow firn segments. There is thus a strong need for alternative independent proxies that record local SMB for modern climatology studies, paleoclimate reconstructions, and ice sheet modeling while avoiding the problems inherent in existing methods.

**Fig. 1:**
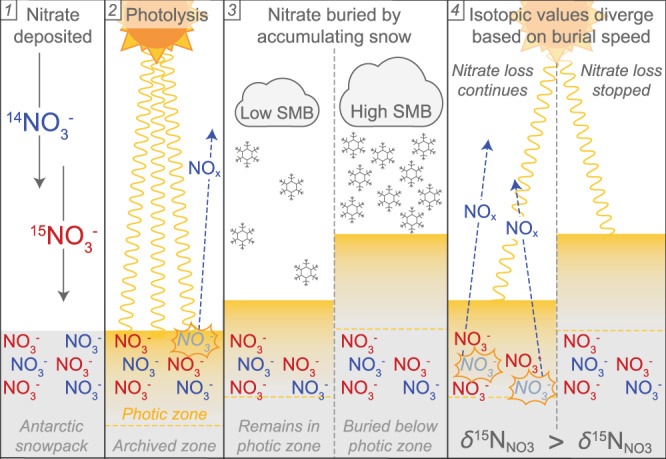
Schematic diagram of the NO_3_^−^ photolytic process in Antarctica. After NO_3_^−^ containing either ^14^N (blue) or ^15^N (red) is deposited on the Antarctic snowpack surface (1), sunlight in the photic zone can trigger photolysis of NO_3_^−^ that favors NO_3_^−^ with a ^14^N atom, which leaves the residual NO_3_^−^ enriched in ^15^N (2). Because sites with lower surface mass balance will accumulate less snow over a given period of time than high surface mass balance sites (3), the NO_3_^−^ at lower surface mass balance sites will remain in the photic zone longer, experience more photolytic mass loss before burial in the archived zone, and have higher *δ*^15^N_NO3arc_ values (4).

Here, we present one such SMB proxy based on photolysis-induced changes in the ^15^N/^14^N ratio (*δ*^15^N, defined as ${\delta }=\frac{{15}_{{{{{{\rm{N}}}}}}}{/{14}_{{{{{{\rm{N}}}}}}}}_{{{{{{\rm{sample}}}}}}}}{{15}_{{{{{{\rm{N}}}}}}}{/{14}_{{{{{{\rm{N}}}}}}}}_{{{{{{\rm{standard}}}}}}}}-1$, relative to the N_2_-air standard) of nitrate (NO_3_^−^) (Fig. 1). Naturally deposited on the Antarctic ice sheet surface as the end product of the atmospheric oxidation of reactive nitrogen^19–22^, NO_3_^−^ within the Antarctic snowpack can be photolytically converted to gaseous nitrogen oxides (NO_x_ = NO + NO_2_) when exposed to ultraviolet light (λ = 290–350 nm). Because ^14^NO_3_^−^ is more readily photolyzed than ^15^NO_3_^−^, the *δ*^15^N of NO_3_^−^ (*δ*^15^N_NO3_) remaining in the snow will increase from its initial depositional value of ≈−20 to +20 ‰ to values as high as +400 ‰^21–28^ as the isotopically lighter photolytic NO_x_ product is ventilated and lost to the atmosphere. Although NO_3_^−^ can also be lost through HNO_3_ volatilization, we interpret *δ*^15^N_NO3_ solely through photolysis since volatilization does not strongly fractionate NO_3_^−^ and should be a very minor component of NO_3_^−^ loss outside of the warmest coastal zones^24,29,30^. Additionally, while the oxygen in NO_3_^−^ also undergoes isotopic fractionation through photolysis, its interpretation is complicated by isotopic interactions with snow and water vapor^24,25,31^ and is not further discussed here.

Photolysis is limited to the depth where light penetrates and initiates photochemical reactions, and so the snowpack can be divided into an uppermost photic zone (generally 10–100 cm in East Antarctica) and a deeper archived zone^31–35^. Photolysis and the resulting isotopic fractionation of NO_3_^−^ cease once snowfall buries NO_3_^−^ beneath the photic zone, and the *δ*^15^N_NO3_ value of the NO_3_^−^ buried in the archived zone (*δ*^15^N_NO3arc_) is assumed to be preserved indefinitely in glacial ice^24,25,31,32^. The final *δ*^15^N_NO3arc_ value reflects the sum total of photolysis inducing radiation experienced by NO_3_^−^ during the burial process, which, assuming stable insolation and photic zone depth, is itself determined by the rate at which the NO_3_^−^ is buried and thus inversely related to SMB^19,25,28,36^. Modeling (Supplementary Discussion 1) and field observations support SMB as the primary driver of spatial variability in *δ*^15^N_NO3arc_ values. Based on a new simplified theoretical framework (Methods, Supplementary Discussion 1), this relationship can be expressed as:

1 $${{{{{\rm{ln}}}}}}({\delta }^{15}{{{{{{\rm{N}}}}}}}_{{{{{{\rm{NO}}}}}}3{{{{{\rm{arc}}}}}}}+1)=\frac{A}{{{{{{\rm{SMB}}}}}}}+B$$

where the regression coefficients *A* and *B* are parameters that subsume constants and linearly co-varying variables associated with photolytic and fractionation processes. The inverse function of Eq. (1) can then be used as a transfer function to reconstruct SMB from *δ*^15^N_NO3arc_ values (SMB_*δ*15N_). Calculated and referenced SMB values are given here with units of kg m^−2^ a^−1^, which is equal to mm w.e. a^−1^.

## Results and discussion

### SMB_*δ*15N_ relationship and spatial applicability

**Fig. 2:**
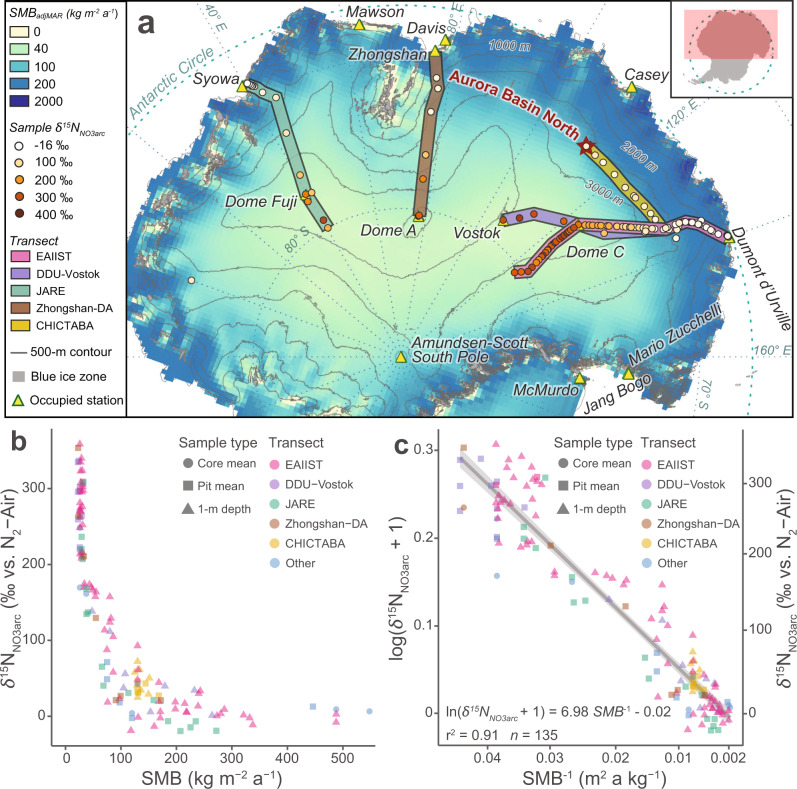
The relationship between Antarctic snow *δ*^15^N_NO3arc_ and surface mass balance (SMB). **a** Map of East Antarctic sites sampled for *δ*^15^N_NO3arc_ along different scientific and logistic transect routes. Colored circles indicate the locations and *δ*^15^N_NO3arc_ values of samples included in our field data set, with *δ*^15^N_NO3arc_ data from the EAIIST (pink) and CHICTABA (yellow) transects newly reported here. The base map SMB data were modeled by MAR^13^ and adjusted for dry site bias (see Methods) with elevation contours from REMA^11^ overlaid. Preservation of NO_3_^−^ is not expected in blue ice zones (gray solid polygons) due to very low or negative SMB and wind scouring^76^. Presently occupied stations in the CONMAP database are shown as labeled triangle icons for spatial reference. **b** Scatter plot of *δ*^15^N_NO3arc_ vs. SMB for all sites in the field dataset. The color of the points corresponds to the transects where the samples were collected as shown in **a**, and the shape of the points corresponds to the sampling method (i.e., snow core, snow pit, or 1-m depth layer). **c** Scatter plot and linear regression of (1) using all sites in the field dataset. The linear regression (gray solid line) is shown with shaded 95% confidence intervals, and regression parameters are displayed at lower left.

To obtain parameter estimates for Eq. (1), we sampled NO_3_^−^ in snow and firn from 92 East Antarctic shallow pits and cores that are reported here. Combined with 43 previously published *δ*^15^N_NO3arc_ samples^23–25,28,31,37^, this constitutes a database of 135 total *δ*^15^N_NO3arc_ values representing 114 distinct sites across East Antarctica (Fig. 2a). These *δ*^15^N_NO3arc_ data were spatially paired with local SMB measurements either observed directly onsite (SMB_ground_) or as an output from the Modèle Atmosphérique Régional (MAR) forced by ERA-interim reanalysis data^13^ and adjusted for a dry-site bias (SMB_adjMAR_) (Methods, Supplementary Discussion 2). The sites in our database cover a comprehensive range of East Antarctic SMB, from 20–30 kg m^−2^ a^−1^ at dome summits on the high plateau to >300 kg m^−2^ a^−1^ for sites on the coastal periphery (Fig. 2b).

The SMB and *δ*^15^N_NO3arc_ in our field dataset are correlated with a high degree of confidence, producing a linear regression where ln(*δ*^15^N_NO3arc_ + 1) = 6.98 ± 0.19 SMB^−1^ − 0.02 ± 0.01 (Fig. 2c, *r*^2^ = 0.91, *p* ≪ 0.001, *n* = 135). Moreover, this relationship is within modeled expectations that use best estimates for photolytic and isotopic fractionation parameters (Supplementary Fig. 1, Supplementary Discussion 1). Although the linear relationship is strong, the spread in regression residuals leads to a relatively large prediction interval of ±0.0085 for each reconstructed SMB_*δ*15N_^−1^ value. This imprecision likely results in part because field sampling techniques varied between studies and best sampling procedures (e.g., well-mixing a > 10 cm layer below the photic zone, taking multiple samples per site) may not always have been followed due to logistical challenges and time constraints. Additionally, the resolution of MAR and other regional climate models cannot capture the impact of small surface features on local SMB, and even hyperlocal SMB variability (i.e., the SMB at scales < 1 m) caused by sastrugi and drifts might be missed by nearby stakes or other ground observations of SMB. Assuming that these factors are not biased toward over- or underestimating SMB, we can expect the SMB_*δ*15N_ regression to provide accurate modeled values despite these prediction intervals. The precision of the regression and its SMB_*δ*15N_ modeled outputs should also improve in the future with the addition of data from new sites using best sampling protocols and improved regional climate modeling.

**Fig. 3:**
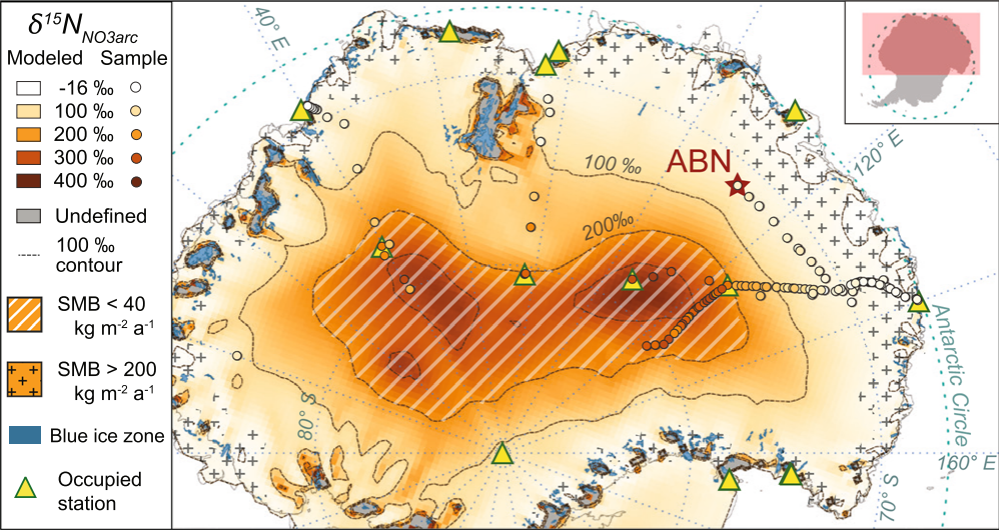
*δ*^15^N_NO3arc_ values modeled by (1) across East Antarctica based on surface mass balance (SMB). The spatial variability of *δ*^15^N_NO3arc_ values across East Antarctica are modeled by applying the field data regression of ln(*δ*^15^N_NO3arc_ + 1) vs. SMB^−1^ to the 1979–2015 mean SMB output (35 km resolution) from the MAR^13^, adjusted for dry site bias (see Methods). Values of *δ*^15^N_NO3arc_ are undefined (gray) at some locations near the coast with very low or negative SMBs due to high sublimation and wind scouring. Preservation of NO_3_^−^ is not expected in these locations, which often correspond to blue ice zones (blue polygons, zones with >100 km^2^ extent shown)^76^. Samples of *δ*^15^N_NO3arc_ from the field database are illustrated by colored circles with the same color gradient as the modeled *δ*^15^N_NO3arc_ values. Regions with SMB less than or greater than 40–200 kg m^−2^ a^−1^ (i.e., the SMB range targeted by the *δ*^15^N_NO3arc_ proxy described here) are illustrated with hatching and crosses, respectively. Presently occupied stations in the CONMAP database are shown as triangle icons for spatial reference, and the Aurora Basin North (ABN) site is indicated with a red star.

Applying the solved regression to SMB values modeled by MAR across East Antarctica reproduces the spatial variability of *δ*^15^N_NO3arc_ observed in samples (Fig. 3, Supplementary Table 3). We find that 74% of Antarctica has *δ*^15^N_NO3arc_ values elevated well above the typical range of atmospheric *δ*^15^N_NO3_ (i.e., >20 ‰), illustrating the vast spatial impact of photolytic NO_3_^−^ loss. The highest modeled values, excluding some small coastal regions with very low or negative modeled SMBs (e.g., the McMurdo Dry Valleys and blue ice zones) where NO_3_^−^ archiving is not expected, are found on the interior high plateau of East Antarctica between Dome C and Dome Fuji, in agreement with previous global chemical transport models^33^. Although millennial-scale changes in global NO_3_^−^ dynamics and atmospheric oxidative capacity are not currently well constrained, the factors parameterized in Eq. (1) (Supplementary Discussion 1) have likely been stable enough during the Holocene for the SMB_*δ*15N_ proxy’s general use. The large changes in atmospheric chemistry, biogeochemical cycles, and global environment earlier in the Pleistocene possibly changed atmospheric NO_3_^−^ isotopic values, snow character, and/or insolation values enough that our SMB_*δ*15N_ proxy based on modern observations will not accurately reconstruct past SMB values in glacial times. However, *δ*^15^N_NO3arc_ changes observed between glacial and interglacial periods in Greenland ice cores have been interpreted to partially record SMB changes^38^, and thus *δ*^15^N_NO3arc_ may still offer important insight into relative changes in SMB and into how NO_3_^−^ dynamics varied during the Pleistocene.

Since the most advanced established technique for NO_3_^−^ isotopic analysis (see Methods) uses ≈5 nmol of NO_3_^−^ for *δ*^15^N_NO3_ analysis and ≈100 nmol to include oxygen isotope anomaly (Δ^17^O_NO3_) analysis, the potential resolution of the SMB_*δ*15N_ proxy depends upon the NO_3_^−^ concentration of the snow or ice sample and upon the mass of snow or ice comprising each sample. For the samples included in our field database, NO_3_^−^ concentrations ranged between 5 and 131 ng g^−1^, with lower values at drier sites. To collect 100 nmol of NO_3_^−^ for maximum isotopic data, these concentrations require between 0.05 to 1.15 kg of snow or ice, with a median requirement of 0.15 kg. For snow pits, sampling at a 2 cm depth interval requires only 0.01–0.16 m^2^ surface area collected per sample, and thus the storage and transport logistics for large numbers of samples are more restrictive for snow pits than physical sampling limitations. Ice core sampling resolution is dependent upon the core diameter and percent of core available for NO_3_^−^ recovery. We find that 2–3 samples per ice core meter are typically achievable even when the ice core is only partly partitioned for NO_3_^−^, and higher resolution is possible with cores that are drilled solely or primarily for NO_3_^−^ isotopic analysis.

While our field dataset covers sites with a SMB from 22 to 548 kg m^−2^ a^−1^, the SMB_*δ*15N_ proxy is best suited for sites with SMB values between 40 and 200 kg m^−2^ a^−1^. Shallow cores from very dry Dome A and Dome C have lower *δ*^15^N_NO3arc_ values at 2–6 m below the surface than at the ~1 m base of the photic zone, possibly because photolytic NO_x_ can be transported downward through firn air convection and re-oxidized into NO_3_^−^ with low *δ*^15^N_NO3_ values (Supplementary Discussion 3). This phenomenon violates the foundational assumption of “locked-in” NO_3_^−^ beneath the photic zone, but we observe it only at the ultra-dry interior sites where SMB < 40 kg m^−2^ a^−1^. For sites with SMB > 200 kg m^−2^ a^−1^, the expected *δ*^15^N_NO3arc_ value falls within the general range of atmospheric *δ*^15^N_NO3_ (−20–+20 ‰) because NO_3_^−^ is buried below the photic zone in less than a year. Despite the short photic zone residence time, more than 80% of NO_3_^−^ is deposited during sunnier months outside of winter polar night^24,39^ and some photolytic loss is still likely. As a result, NO_3_^−^ samples that integrate multiple years of accumulation at high SMB sites might still resolve differences in SMB (Supplementary Fig. 3). Additionally, *δ*^15^N_NO3arc_ values are increasingly less sensitive to SMB changes with higher SMB values due to the asymptotic nature of SMB^−1^ (i.e., the relationship between *δ*^15^N_NO3arc_ and SMB is nearly flat where SMB > 200 kg m^−2^ a^−1^ as seen in Fig. 2b). Despite these restrictions, over 59 % of Antarctica has a SMB between 40 and 200 kg m^−2^ a^−1^^13^ (Fig. 2a), and additional study of NO_3_^−^ dynamics in wet and dry extremes may reveal regional adjustments that allow for further application of this new SMB proxy (Supplementary Discussion 4, Supplementary Fig. 3).

### Aurora Basin North SMB reconstruction

**Fig. 4:**
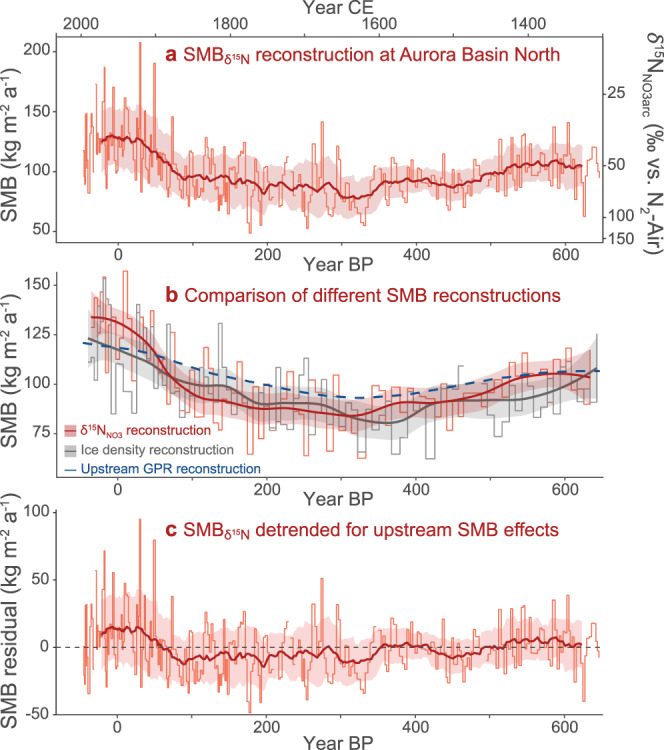
Reconstructions of surface mass balance (SMB) for an Antarctic ice core from ABN. **a** SMB for Aurora Basin North based on *δ*^15^N_NO3arc_ data from the ABN1314-103 ice core. Reconstructed SMB_*δ*15N_ values are shown by the red stepped lines with the 50-yr running mean±1σ overlaid as a darker thick line and shaded zone. **b** Comparison of SMB values reconstructed from *δ*^15^N_NO3arc_ (red) with those from ice density (gray) and upstream GPR isochron depth^48^. The SMB_*δ*15N_ and SMB_GPR_ values were aggregated to match the 1-m resolution of the SMB_density_ data. For SMB_*δ*15N_ and SMB_density_, smoothed LOESS curves are overlaid to more clearly show long-term patterns. **c** SMB_*δ*15N_ values after the upstream topographic impact on SMB has been removed, with 50-yr running mean±1σ values overlaid. The resulting residuals may better illustrate SMB variability due to climate change.

As a proof of concept, we applied the SMB_*δ*15N_ transfer function to *δ*^15^N_NO3arc_ data from the 103 m deep ABN1314-103 ice core. This core was one of three drilled in the Australian Antarctic Program’s 2013–2014 summer campaign at Aurora Basin North (ABN; 71.17 °S 111.37 °E, 2679 m above sea level), a site with moderate modern SMB (≈120 kg m^−2^ a^−1^) located midway between coastal Casey Station and the Dome C summit (Fig. 2a). The SMB_*δ*15N_ history reconstructed from ABN1314-103 covers the period from −47 to 649 years before present (BP, where present = 1950 CE) and has values ranging from 49 to 208 kg m^−2^ a^−1^ (Fig. 4a). Each SMB_*δ*15N_ value integrates an average of 2.4 years of accumulation (total range: 0.7–4.5 years), and thus any impacts from individual precipitation events or seasonal extremes are attenuated. Overall, the SMB values at this site show fairly large variability (coefficient of variation = 0.21). The mean SMB_*δ*15N_ in the 20^th^ century (126 ± 26.5 kg m^−2^ a^−1^) is 34% greater than the mean SMB_*δ*15N_ before 1900 CE (94 ± 18 kg m^−2^ a^−1^) and nearly 52% greater than the driest century that spans the 1600s CE (83 ± 20 kg m^−2^ a^−1^) (Fig. 4a).

Since *δ*^15^N_NO3arc_ values reflect the snow burial speed of the immediate overlying area, short-term variability in SMB_δ15N_ is likely dominated by small spatial scale factors such as surface roughness (e.g., sastrugi and dune migration)^40–43^ and local weather (e.g., snowfall heterogeneity)^12,43–45^. However, the SMB_*δ*15N_ patterns observed over decadal to centennial scales more likely represent changes to the broader regional environment as the local environmental “noise” has less impact when data is aggregated at longer timescales. Finally, it is important to note that the SMB_*δ*15N_ values reflect the immediate local snow accumulation, and so some short duration events (e.g., atmospheric rivers) with major region-spanning impacts may not be preserved in an individual ice core due to periods of surface erosion and/or mixing^46^. This feature should not, however, be viewed as a drawback of the SMB_*δ*15N_ proxy. Rather, the SMB_*δ*15N_ record is accurately reflecting the actual SMB experienced at the core site, which is a critical factor to accurately calculating and interpreting other environmental proxies contained in the ice core, such as biogeochemical fluxes.

### Validating the SMB_*δ*15N_ proxy reconstruction

We verified our new proxy’s accuracy by comparing the SMB_*δ*15N_ values with SMB calculated using the physical density of the ice core and its age-depth relationship (SMB_density_). Because the measurements for SMB_density_ are typically performed on each individual ice core segment, it generally has a lower potential resolution than SMB_*δ*15N_ which, in contrast, can have multiple values per core segment. Still, SMB_density_ functions well as an established benchmark for validating newer SMB proxies like SMB_*δ*15N_. For each 1-m core segment of ABN1314-103, we calculated a SMB_density_ value by dividing the segment’s mass (kg) by both its volume (m^3^) and the age difference between the top and bottom of the segment (a m^−1^). The SMB_*δ*15N_ (aggregated to match the 1-m resolution) and SMB_density_ share very similar mean values (100.8 vs. 98.0 kg m^−2^ a^−1^, respectively) and total SMB ranges (62.0–157.3 vs. 61.7–153.4 kg m^−2^ a^−1^, respectively), and the two SMB reconstructions have a similar pattern of variation with a moderate Pearson correlation (*r* = +0.46, *p* < 0.001, *n* = 90) (Fig. 4b). The correlation increases rapidly when a broader running average is applied to the data, reaching +0.72 with 25 year averaging and +0.82 with 50 year averaging. This agreement in mean value, range, and variability validates our SMB_*δ*15N_ approach and the potential of *δ*^15^N_NO3arc_ as an accurate proxy for paleoenvironmental change.

Interpreting the ABN1314-103 SMB profile is more complicated than for ice cores drilled at dome summits because the ice sheet at the ABN drilling site is flowing horizontally at a rate of 16.2 m a^−1^^47^. This means that the ice in ABN1314-103 actually accumulated as snow along a continuous 11.5 km transect upstream of the current ABN drilling site, with the oldest and deepest ice originating from the most distant upstream position. Using the horizontal ice flow rate and the ABN1314-103 core’s age-depth model, we can estimate the position along the upstream transect where the snow for each depth in the core originally accumulated^48^.

**Fig. 5:**
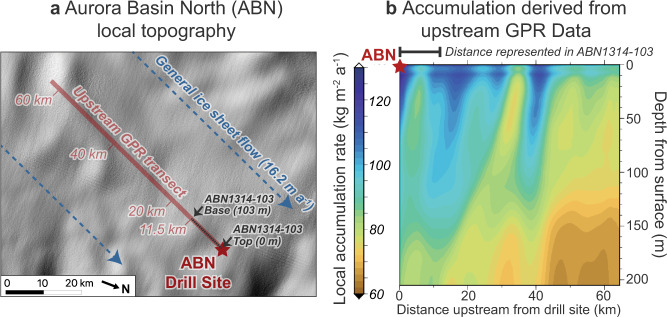
Topography and accumulation patterns upstream of the Aurora Basin North (ABN) drill site. **a** Local surface topography of the ice sheet around the ABN ice core drilling site, shown as a hillshade derived from the REMA digital elevation model^11^ with 100x vertical exaggeration. Ground-penetrating radar measurements were taken along a 60 km transect upstream of the drill site relative to local ice sheet flow, and the ice contained in the ABN1314-103 core corresponds to the first 11.5 km of the transect. **b** Local accumulation rate variability with depth along the upstream ABN transect determined from radar identification of isochronal internal reflective horizons, reflecting past changes in surface mass balance. Regions of relatively higher or lower accumulation preserved with depth likely represent the influence of long-lived surface topographic features. Accumulation rates have an original depth resolution of 0.5 m which is smoothed through a moving age-depth average with a cosine weighting window to reduce isochron artifacts^49^.

Although overall elevation gain is small along the 11.5 km transect (<15 m), the region has abundant 0.5–1 m undulations in surface topography extending over horizontal extents of 3–10 km^11^ (Fig. 5a). The MAR’s horizontal grid size (35 km) cannot resolve any potential SMB impact from these features, but ground penetrating radar (GPR)^49^ data collected along the upstream transect reveals that these surface slope and curvature changes correlate with SMB variations of up to 40 kg m^−2^ a^−1^ as determined by internal isochronal radar reflection horizons^48^ (Fig. 5b). These surface features can be identified as buried horizons to depths below the deepest segment of ABN1314-103, which suggests that they have been stable features of the local landscape for at least 700 years.

Because ABN1314-103 is composed of snow that fell along this upstream transect, the ice core SMB record will not only reflect changes due to wetting or drying of the regional climate, but it will also reflect any spatial SMB variability caused by topographic features that existed along the upstream transect. Since the local surface topography has not significantly shifted or changed over the time period covered by ABN1314-103, we take the modern topography-driven SMB changes observed with GPR to be representative of past SMB spatial variability. As each position along the upstream transect is paired to a depth in ABN1314-103, we can transfer the GPR-derived SMB profile along the horizontal transect to ice core depths to produce a SMB reconstruction (SMB_GPR_) that can be directly compared to the SMB_density_ and SMB_*δ*15N_ reconstructions.

The SMB_GPR_ reconstruction for ABN1314-103 (Fig. 4b) represents the component of the SMB record preserved in the ice core that can be explained by upstream surface topography alone. We find that the general pattern of variability in SMB_GPR_ correlates very well with the patterns recorded in the SMB_*δ*15N_ (*r* = +0.74) and SMB_density_ (*r* = +0.63) records (Fig. 4b). Thus, it appears that the primary SMB pattern preserved in ABN1314-103 is driven by upstream changes in surface curvature, which is important for properly interpreting other environmental proxies contained in the ice and for understanding the local ice flow history.

### Extracting a climate-driven SMB record

To examine whether a secondary signal related to climate change was also preserved by the *δ*^15^N_NO3arc_, we removed the spatial impact of upstream topography by subtracting the SMB_GPR_ data from the SMB_*δ*15N_ record. After this “upstream effect detrending” and accounting for a small consistent offset in mean SMB values (3.7 kg m^−2^ a^−1^) between SMB_GPR_ and SMB_*δ*15N_, we find that the multi-decadal SMB values have been generally stable over the past 700 years (Fig. 4c), with 50-yr running averages of the SMB always within 15 kg m^−2^ a^−1^ from the mean of the detrended data. These running averages suggest that drier conditions existed at ABN between 60 and 350 yr BP (1600 and 1890 CE, partially corresponding to the Little Ice Age) and that precipitation has increased in the most recent 100–150 years. This is generally consistent with what has been observed at other East Antarctic sites^50–53^ and for Antarctica as a whole^18^, but we recognize that this pattern is similar to the upstream topographic effect and that it might also arise if the SMB_GPR_ record is excessively smoothed relative to true topographic-driven SMB variability (perhaps by the GPR data processing).

On shorter timescales, SMB values frequently change by ≈50 kg m^−2^ a^−1^ around a common mean within 10–20 year periods. This pattern likely reflects the high interannual snowfall variability expected at sites like ABN^14^. Located at the transition between the coast and the interior East Antarctic Plateau, annual snow accumulation at ABN is sensitive to frequent intrusions of extreme precipitation events and atmospheric rivers^44,45^, and the observed sub-decadal SMB_*δ*15N_ variability may represent the frequency of their stochastic occurrence at the site. Additionally, small scale surface roughness features like sastrugi may affect hyperlocal SMB through periods of enhanced accumulation and erosion as they migrate and evolve on the snow surface^40–42,54^. While the temporal evolution and possible life cycle cyclicity of surface roughness features are as yet poorly known, hyperlocal changes in SMB could also explain some of the short-term SMB variability observed in the ABN record if the sampling interval is shorter than the average duration of a surface feature at a given location.

### Applied use and potential of the SMB_*δ*15N_ proxy

With over 8 million km^2^ of Antarctica having a SMB between 40 and 200 kg m^−2^ a^−1^^13^ and over 70% of the ice sheet area modeled to have *δ*^15^N_NO3_ values markedly elevated by photolysis, the SMB_*δ*15N_ proxy holds great potential for expanding our knowledge of Antarctic SMB variability over time and space and serving as an independent supplemental SMB reconstruction. Currently, regions with moderate SMB have only a handful of sites with SMB records older than 200 years, with the East Antarctic Plateau particularly poorly represented^18^. For ice coring projects in these regions, the SMB_*δ*15N_ proxy can perform better at capturing the local effects of strong winds, irregular surface topography, and high interannual snowfall variability than water isotopic techniques while avoiding problems with layer thinning, density modeling, and core damage that affect density-based methods. As regional climate models still struggle to accurately simulate drifting snow and sublimation fluxes in the coast-to-plateau transition^13^, SMB_*δ*15N_ can provide critical ground-based data for models predicting future contributions to sea level rise. The SMB_*δ*15N_ proxy also holds particular value for helping to constrain and validate models of upstream flow effects in research targeting ice streams and broad-scale glacial flow patterns. The SMB_*δ*15N_ approach may also be useful to estimate relative SMB changes for ice cores that lack robust age-depth models due to severe glacial deformation or discontinuities.

Additionally, sampling for the SMB_*δ*15N_ proxy can save valuable time and cost compared to existing alternatives to expand current records of modern SMB. Obtaining new ground-based SMB measurements using existing techniques for sites without annually resolved layers requires either coring several meters to the increasingly buried Pinatubo volcanic horizon or repeated visits to newly installed stake transects. However, limited time and resources for research expeditions to remote areas precludes intensive SMB surveys with these methods. With the SMB_*δ*15N_ proxy, a mean site SMB could be determined with only a series of shallow snow or firn samples extending deep enough into the archived zone to cover only a few seasonal cycles (much shallower than the Pinatubo horizon). After mixing snow well from multiple samples, only 15–75 g (0.3–1.5 kg if Δ^17^O_NO3_ results are desired) would need to be kept, transported, and analyzed for each sample, which logistically allows for the rapid collection of robust SMB site means in many locations. On-site melting and NO_3_^−^ concentration could further reduce logistical requirements.

The SMB_*δ*15N_ proxy promises to grow and adapt as studies on Antarctic NO_3_^−^ dynamics continue. More NO_3_^–^ samples coupled with quality environmental context data from East Antarctic will help us better constrain the uncertainty of SMB_*δ*15N_ calculations and allow for more confidence in reconstructions. As additional ice cores are analyzed for *δ*^15^N_NO3arc_, we can better understand under which exact conditions *δ*^15^N_NO3arc_ most accurately records SMB variability and if we can improve our reconstructions with a more complex model. Differences between calculated SMB_*δ*15N_ values and well-constrained SMB_density_ values may also prove useful in identifying periods of unusual environmental conditions that alter typical photolytic reactivity.

Because the resolution of *δ*^15^N_NO3arc_ sampling is limited only by the minimum amount of NO_3_^−^ needed for analysis, very finely-resolved *δ*^15^N_NO3arc_ records can be obtained by increasing the mass of ice collected per depth unit (e.g., by specifically drilling whole cores or replicate cores for NO_3_^−^ isotopes) and with advances in NO_3_^−^ isotopic analysis expected in the near future^55^. This may allow for more precise multi-annual aggregations for SMB_*δ*15N_ reconstructions and permit a deeper examination of subannual NO_3_^−^ dynamics that can improve the proxy. Given the potential of the SMB_*δ*15N_ proxy to advance our understanding of the Antarctic environment and its sensitivity to climate change, we strongly recommend that potential ice coring projects incorporate NO_3_^−^ analyses into their planning and urge continued studies on Antarctic NO_3_^−^ dynamics.

## Methods

### Mathematical framework for δ^15^N_NO3arc_ and SMB relationships

A linear relationship between *δ*^15^N_NO3arc_ and the reciprocal of surface mass balance (*SMB*^−1^) has been previously observed and reported in Antarctica^19,28,36^. Here, we mathematically illustrate how this relationship between *δ*^15^N_NO3arc_ and SMB arises through photolysis of NO_3_^−^. We focus solely on the characteristics of NO_3_^−^ contained within a given horizontal plane of snow that is located at the snowpack surface at *t* = 0. We assume simplified conditions with a constant surface mass balance (*SMB*), clear sky conditions, no surface roughness, and no significant compaction with burial in the photic zone. Any NO_3_^−^ that is photolyzed is immediately and permanently removed from the plane of snow, and NO_3_^−^ recycling^31,36^ is assumed not to affect NO_3_^−^ in the plane of snow during the burial process modeled here (i.e., after *t* = 0).

#### Defining the relationship between *δ*^15^N_NO3arc_ and SMB

The time that it takes for a given horizontal plane of snow to be buried from the surface to a particular depth *z* is determined by the SMB (kg m^−2^ a^−1^, converted to an equivalent vertical velocity in cm s^−1^):

2 $${t}_{(z)}=\frac{z}{{{{{{\rm{SMB}}}}}}}$$

The concentration of NO_3_^−^ within a plane of snow decays through time according to:

3 $$\frac{d[{{{{{{{\rm{NO}}}}}}}_{3}}^{-}]}{{dt}}=-{J}_{(z)}{[{{{{{{{\rm{NO}}}}}}}_{3}}^{-}]}_{(t)}$$

where *J*_*(z)*_ is the photolytic rate constant at a given depth defined as:

4 $${J}_{(z)}=\sigma {{\upphi }}{I}_{(z)}$$

where *σ* is the absorption cross section for NO_3_^−^ photolysis (cm^2^), *ɸ* is the quantum yield for NO_3_^−^ photolysis (molec photon^−1^), and *I*_*(z)*_ is the actinic flux of ultraviolet irradiance (photon cm^−2^ s^−1^ nm^−1^) integrated over wavelengths that can induce photolysis of NO_3_^−^. However, this photolytic rate “constant” changes with depth because actinic flux exponentially decays with depth as:

5 $${I}_{(z)}={I}_{0}{e}^{\frac{-z}{{z}_{e}}}$$

where *I*_*0*_ is the initial actinic flux that strikes the snow surface and *z*_*e*_ is the *e*-folding depth (cm) of the snowpack. Note that non-exponential decay of *I* in the top ~2 cm of snowpack^32^ is simplified here by assuming the decay to be exponential from the snow surface. Equation (3) can then be expressed as:

6 $$\frac{d[{{{{{{{\rm{NO}}}}}}}_{3}}^{-}]}{{dt}}=-\sigma {{\upphi }}\,{I}_{o}{e}^{\frac{-z}{{z}_{e}}}{[{{{{{{{\rm{NO}}}}}}}_{3}}^{-}]}_{(t)}$$

Through Eq. (2), we can rewrite Eq. (6) as:

7 $$\frac{d[{{{{{{{\rm{NO}}}}}}}_{3}}^{-}]}{{dt}}=-\sigma {{\upphi }}\,{I}_{o}{e}^{\frac{-{{{{{\rm{SMB}}}}}}t}{{z}_{e}}}{[{{{{{{{\rm{NO}}}}}}}_{3}}^{-}]}_{(t)}$$

In order to determine the NO_3_^−^ concentration at a given depth, we use the relationship between depth and time (*z* = SMB × *t*) to derive:

8 $$\frac{d[{{{{{{{\rm{NO}}}}}}}_{3}}^{-}]}{{[{{{{{{{\rm{NO}}}}}}}_{3}}^{-}]}_{(t)}}=-\sigma {{\upphi }}\,{I}_{o}{e}^{\frac{-{{{{{\rm{SMB}}}}}}t}{{z}_{e}}}{dt}$$

And integrate to produce:

9 $${{{{{\rm{ln}}}}}}{[{{{{{{{\rm{NO}}}}}}}_{3}}^{-}]}_{\left(t\right)}=\frac{\sigma {{\upphi }}\,{I}_{o}\,{z}_{e}\,{e}^{\frac{-{{{{{\rm{SMB}}}}}}t}{{z}_{e}}}}{{{{{{\rm{SMB}}}}}}}+C$$

Which simplifies to:

10 $${[{{{{{{{\rm{NO}}}}}}}_{3}}^{-}]}_{\left(t\right)}={{e}^{c}e}^{\frac{\sigma {{\upphi }}{I}_{o}{z}_{e}{e}^{\frac{-{{{{{\rm{SMB}}}}}}t}{{z}_{e}}}}{{{{{{\rm{SMB}}}}}}}}$$

At *t* = 0, [NO_3_^−^]_(t)_ = [NO_3_^−^]_0_ and therefore:

11 $${e}^{c}={[{{{{{{{\rm{NO}}}}}}}_{3}}^{-}]}_{0}\,{e}^{\frac{-\sigma {{\upphi}}{I}_{o}{z}_{e}}{{{{{{\rm{SMB}}}}}}}}$$

And thus combining Eqs. (10) and (11):

12 $${[{{{{{{\rm{NO}}}}}}_{3}}^{-}]}_{\left(t\right)}={{[{{{{{{{\rm{NO}}}}}}}_{3}}^{-}]}_{0}{e}^{\frac{-\sigma {{\upphi }}{I}_{o}{z}_{e}}{{{{{{\rm{SMB}}}}}}}}e}^{\frac{\sigma {{\upphi }}{I}_{o}{z}_{e}{e}^{\frac{-{{{{{\rm{SMB}}}}}}t}{{z}_{e}}}}{{{{{{\rm{SMB}}}}}}}}={{[{{{{{{{\rm{NO}}}}}}}_{3}}^{-}]}_{0}{e}}^{\frac{\sigma {{\upphi }}{I}_{o}{{z}}_{e}({e}^{\frac{-{{{{{\rm{SMB}}}}}}t}{{z}_{e}}}-1)}{{{{{{\rm{SMB}}}}}}}}$$

According to Eq. (12), as time (i.e., burial depth) increases, the NO_3_^−^ concentration will decrease. However, the rate of decrease will lessen over time as the value of *SMB × t* approaches 3*z*_*e*_ and 95% of the initial irradiance is gone. Here, below the photic zone (i.e., *z* > 3*z*_*e*_), the NO_3_^−^ concentration is largely stable and equal to *e*^*c*^.

Therefore, we can calculate the fraction of NO_3_^−^ archived below the photic zone (*f*_*NO3arc*_) as:

13 $${f}_{{{{{{\rm{N}}}}}}{{{{{{\rm{O}}}}}}3}_{{{{{{\rm{arc}}}}}}}}=\frac{{e}^{c}}{{[{{{{{{{\rm{NO}}}}}}}_{3}}^{-}]}_{0}}=\frac{{{[{{{{{{{\rm{NO}}}}}}}_{3}}^{-}]}_{0}{e}}^{\frac{-\sigma {{\upphi }}{I}_{o}{z}_{e}}{{{{{{\rm{SMB}}}}}}}}}{{[{{{{{{{\rm{NO}}}}}}}_{3}}^{-}]}_{0}}={e}^{\frac{-\sigma {{\upphi }}{I}_{o}{z}_{e}}{{{{{{\rm{SMB}}}}}}}}$$

To determine the *δ*^15^N_NO3arc_ of this NO_3_^−^, Rayleigh fractionation states that *δ*^15^N_NO3_ can be calculated with the fractionation factor *ɑ* by:

14 $${{{{{\rm{ln}}}}}}\left({{\delta }}^{15}{{{{{{\rm{N}}}}}}}_{{{{{{{\rm{NO}}}}}}3}_{{{{{{\rm{arc}}}}}}}}+1\right)=\left(a-1\right){{{{{\rm{ln}}}}}}\left({f}_{{{{{{\rm{N}}}}}}{{{{{{\rm{O}}}}}}3}_{{{{{{\rm{arc}}}}}}}}\right)+{{{{{\rm{ln}}}}}}\left({{\delta }}^{15}{{{{{{\rm{N}}}}}}}_{{{{{{{\rm{NO}}}}}}3}_{0}}+1\right)$$

Through our prior calculation of *f*_*NO3arc*_ in Eq. (13), we thus produce:

15 $${{{{{\rm{ln}}}}}}\left({{\delta }}^{15}{{{{{{\rm{N}}}}}}}_{{{{{{{\rm{NO}}}}}}3}_{{{{{{\rm{arc}}}}}}}}+1\right)=\left(a-1\right)\frac{-\sigma {{\upphi }}\,{I}_{o}\,{z}_{e}}{{{{{{\rm{SMB}}}}}}}+{{{{{\rm{ln}}}}}}\left({{\delta }}^{15}{{{{{{\rm{N}}}}}}}_{{{{{{{\rm{NO}}}}}}3}_{0}}+1\right)$$

Because (ɑ − 1) is negative for nitrogen during photolysis of NO_3_^−^^23,24,33,56–58^ and the other parameters are positive, this means that *δ*^15^N_NO3arc_ will vary linearly and positively with *SMB*^−1^ when other parameters are held constant or scale linearly with SMB^−1^. We examine the potential impacts of variability in these other parameters more thoroughly in Supplementary Discussion 1.

Based on modeling and field observations, SMB is the primary driver of change in *δ*^15^N_NO3arc_ values. Thus, the non-SMB variables can be subsumed into two parameters *A* and *B* to function as linear regression coefficients, producing Eq. (16) of the main text:

16 $${{{{{\rm{ln}}}}}}\left({{\delta }}^{15}{{{{{{\rm{N}}}}}}}_{{{{{{{\rm{NO}}}}}}3}_{{{{{{\rm{arc}}}}}}}}+1\right)=\frac{A}{{{{{{\rm{SMB}}}}}}}+B$$

The inverse function of Eq. (16) can be used as a transfer function to calculate SMB based on a *δ*^15^N_NO3arc_ value:

17 $$\frac{1}{S{{{{{\rm{MB}}}}}}}=\frac{{ln}\left({\delta }^{15}{{{{{{\rm{N}}}}}}}_{{{{{{\rm{NO}}}}}}3{{{{{\rm{arc}}}}}}}+1\right)-B}{A}$$

Finally, since ln(x + 1) ≈ x when x ≈ 0, a simpler relationship of Eq. (15) can be approximated, in a form similar to that previously reported from field observations^25,28,36^:

18 $${{\delta }}^{15}{{{{{{\rm{N}}}}}}}_{{{{{{{\rm{NO}}}}}}3}_{{{{{{\rm{arc}}}}}}}}=\left(a-1\right)\frac{-\sigma {{\upphi }}\,{I}_{o}\,{z}_{e}}{{{{{{\rm{SMB}}}}}}}+{{\delta }}^{15}{{{{{{\rm{N}}}}}}}_{{{{{{{\rm{NO}}}}}}3}_{0}}$$

### Snow sampling techniques

The *δ*^15^N_NO3arc_ values in our database are taken from a mix of previously reported values from Antarctic research traverses and values newly reported here (Fig. 2). For all values, snow and ice containing NO_3_^−^ was sampled in the field in one of three techniques: 1) 1–2 m deep snow pit with continuous sampling at regular intervals from top to bottom, 2) single sample taken of a well-mixed 5–10 cm layer around the 1-m depth layer, and 3) drilled core later cut at desired intervals. For isotopic measurement of NO_3_^–^ that included Δ^17^O_NO3_ analysis, 0.3–1.5 kg of snow or ice per sample were gathered to ensure a sufficient amount of NO_3_^−^. Generally, the multiple samples produced by the snow pit technique offered the best and most flexible results, but the 1-m depth layer technique was valuable for quick sampling during limited stops, and cores are necessary to collect samples deeper than ≈5 m.

### Laboratory analyses

For *δ*^15^N_NO3arc_ results included in our database that have been previously reported, readers are directed to the original papers for specific analytical and sampling techniques. For the *δ*^15^N_NO3arc_ data newly reported here, snow and ice samples were collected into clean sealed plastic bags or tubs and stored frozen until melted at room temperature for analysis. The NO_3_^−^ mass fraction (*ω*(NO_3_^−^)) was determined on aliquots by either a colorimetric method or ion chromatography with detection limits <0.5 ng g^−1^ and precision of <3 %^23,24^. The remaining melted samples were passed through an anionic exchange resin (Bio-Rad™ AG 1-X8, chloride form), and the resulting trapped NO_3_^−^ was eluted with 10 ml of NaCl 1 M solution.

Isotopic analysis occurred at IGE-CNRS, Grenoble, France, where NO_3_^−^ in these samples was converted to N_2_O with the denitrifying bacteria *Pseudomonas aureofaciens* (lacking nitrous oxide reductase), thermally decomposed into O_2_ and N_2_ on a 900 °C gold surface, and separated by gas chromatography with a GasBench II™. Oxygen and nitrogen isotopic ratios were then measured on a Thermo Finnigan™ MAT 253 mass spectrometer^59–62^. Isotopic effects from this analysis were corrected^23,60^, using the international reference materials USGS 32, USGS 34, and USGS 35 with ultrapure Dome C water used for standards and samples throughout the analyses to account for potential oxygen isotopic exchanges. Results are reported relative to Vienna Standard Mean Ocean Water (V-SMOW) for oxygen isotopes^63^ and N_2_-Air for nitrogen isotopes^64^.

For snow pits with multiple sequential *δ*^15^N_NO3arc_ values, a single *δ*^15^N_NO3arc_ value was calculated as the aggregate of samples 30+ cm deep, weighted by the relative mass of NO_3_^−^ per sample. Although the photic zone boundary can extend lower than 30 cm at some sites^31,32^, this cutoff was deemed an acceptable compromise to include more data from pits that stopped at 50 cm depth as the great majority of photolysis will have occurred within the top 30 cm due to exponential decay of actinic flux and *ω*(NO_3_^−^) with depth. Exceptions to this were made for three coastal pits from Cap Prud’homme (weighted-means of 3+ cm samples), where high accumulation greatly reduces photolytic impact, higher snow impurities reduce the photic zone depth, and a broader aggregation is necessary to smooth seasonal cycles. Additionally, two pits from Dronning Maud Land were aggregated with 15+ cm samples based on shallow 3*z*_*e*_ values (2–5 cm) calculated on site during snow pit sampling^31^. For cores included in our database, a single *δ*^15^N_NO3arc_ value to be considered representative of the site was calculated as the isotopic mean of samples extending from present back to no earlier than 1800 CE.

Noro et al. (2018) reported *δ*^15^N_NO3_ values for 16 pits along the JARE54 and JARE57 transects^28^, but the sampling methodology for these pits took a single well-mixed sample of the entire pit depth which included the entire photic zone. In order to estimate the *δ*^15^N_NO3arc_ values of these sites (i.e., the value as if the photic zone snow had been excluded), we applied a correction factor calculated using data from other pits in our database that were taken on two similar transects spanning from the coast to other interior domes (Dome A and Dome C) of East Antarctica^24,25^. Because each of the pits on the Dome A and Dome C transects were continuously sampled at discrete intervals from the surface to a point below the photic zone, we calculated different weighted-mean *δ*^15^N_NO3_ values for selected depth spans that matched the three extents of the JARE pits: 0–30 cm, 0–50 cm, and 0–80 cm. Corrective factors were calculated through the linear regression of *δ*^15^N_NO3arc_ vs. δ^15^N_NO3.X_ from Dome A/Dome C transect pits (where *δ*^15^N_NO3arc_ is our database’s *δ*^15^N_NO3_ value from the archived zone and δ^15^N_NO3.X_ is the weighted-mean value of samples from the surface to depth *x*: 30, 50, or 80 cm) and applied to the JARE pit data through the appropriate depth correction (Supplementary Tables 4, 5). Corrections were not made for JARE samples where *δ*^15^N_NO3_ < 0 ‰, as these low *δ*^15^N_NO3_ values strongly suggest that photolysis was not a significant factor at these coastal sites, and photic zone corrections were thus not warranted.

### SMB data

In our database, 74 *δ*^15^N_NO3arc_ samples are represented by 51 unique direct ground measurements of SMB (SMB_ground_) values observed at or near the NO_3_^−^ sampling site, with the numerical discrepancy due to some sites having replicate *δ*^15^N_NO3arc_ samples. These previously reported SMB_ground_ values were determined by measuring the change in surface height on established stakes or poles, by measuring the mass between known volcanic or radioactivity horizons in an ice core, or by GPR identification of dated horizons^10,12,24,25,65–71^.

Regional climate models can be used to estimate modern SMB rates for sites lacking ground observations^7,13^, and we used the Modèle Atmosphérique Régional (MAR) version 3.6.4 with European Centre for Medium-Range Weather Forecasts “Interim” re-analysis data (ERA-interim) data as applied by Agosta et al. (2019) to model mean annual SMB at all database sites for the period 1979–2017^13^. Because the MAR overestimates SMB at high elevation (>3000 m) interior sites of the East Antarctic plateau^72^, we calculated a correction factor through linear regressions of SMB_ground_ vs. MAR-estimated SMB (SMB_MAR_) for our 51 sites that have both values (Supplementary Table 6, Supplementary Fig. 4). This correction was applied to all original MAR estimates to produce “adjusted-MAR” SMB (SMB_adjMAR_) that match more closely with ground observations.

These sites were then split into two overlapping subsets of roughly equal count (SMB_MAR_ < 175 kg m^−2^ a^−1^ and SMB_MAR_ is >110 kg m^−2^ a^−1^), and a linear regression was calculated for each subset of sites. This regression for sites where SMB_MAR_ < 175 kg m^−2^ a^−1^ is tightly constrained (SMB_ground_ = 1.0 ± 0.1 × SMB_MAR_ − 5.8 ± 7.1, *r*^2^ = 0.84), and it performs well to align the SMB_MAR_ estimates with the SMB_ground_ values at low SMB sites. The subset of SMB_MAR_ is >110 kg m^−2^ a^−1^ has some samples where the difference between SMB_MAR_ and SMB_ground_ are very large, particularly at lower elevation sites where intense aeolian erosion and deposition often produce highly variable local SMB rates that are difficult to accurately model^13,14^. As a result, this regression is weaker (SMB_ground_ = 0.9 ± 0.2 × SMB_MAR_ + 4.2 ± 57.9, *r*^2^ = 0.35) than the first regression, but we apply it while acknowledging the possibility of wide deviations. The two regressions intersect at (SMB_MAR_ = 138 kg m^−2^ a^−1^, SMB_ground_ = 130 kg m^−2^ a^−1^), and thus SMB_adjMAR_ values were calculated by applying the first regression to all sites where SMB_MAR_ ≤ 138 kg m^−2^ a^−1^ and applying the second regression to all sites where SMB_MAR_ > 138 kg m^−2^ a^−1^. We constructed our final primary SMB dataset for the analysis of *δ*^15^N_NO3arc_ samples by using the best quality SMB data for each site: SMB_ground_ if available and SMB_adjMAR_ otherwise.

### Transfer function and SMB reconstruction

We modeled linear relationships between ln(*δ*^15^N_NO3arc_ +1) and SMB^−1^ based on Eq. (15) using previously reported parameter values to compare our theoretical framework to field results and to better understand the sensitivity of the relationships to photolytic and fractionation factors (Supplementary Discussion 1). To determine the coefficients in Eq. (1) from our field data, we performed linear regressions using all database *δ*^15^N_NO3arc_ samples and the primary SMB dataset of best available SMB. Additional regressions (Supplementary Discussion 2) were performed for subsets of the database based on SMB type (SMB_ground_ vs. SMB_adjMAR_). With regression coefficients determined for Eq. (1), we modeled the spatial distribution of *δ*^15^N_NO3arc_ values across Antarctica using gridded mean SMB (MAR-ERA-interim, 1979–2015) at a 35 km resolution^13^ that were converted to SMB_adjMAR_ as previously described.

For reconstructing the ABN SMB_*δ*15N_ history, the ABN1314-103 ice core was cut into 0.33 m samples from 5 to 103 m, and these were processed for NO_3_^−^ isotopes in 2016 as previously described. We applied an annually resolved age model (ALC01112018) based on seasonal ion and water isotope cycles and constrained by volcanic horizons that was originally developed for a longer core also taken at ABN. Each 1 m ice core segment was individually weighed prior to cutting, and the mass and volume were used to calculate a SMB profile based on dated ice density changes (SMB_density_).

To determine past topographical effects on SMB, a MALA GPR device towing a RTA antenna on the surface (50 MHz out, 100 MHz in) was operated for a 65 km transect upstream of the coring site as part of the 2013–2014 campaign. Radar was triggered every 2 s (i.e., every 6–7 m along the transect) with a recording time window of 3000 nanoseconds that captured returns down to 300 m depth. After postprocessing^49^, isochronal internal reflecting horizons were identified to 220 m depth, digitized with ReflexW software, and dated by connecting to the ALC01112018 age-depth model. Using a density profile taken from a longer ice core simultaneously drilled at ABN, 2D fields (depth by transect distance) were calculated for age, mean accumulation rate, and local accumulation rate. The mean accumulation rate to the most shallow reflecting horizon was taken as the upstream topographical effect on SMB (i.e., SMB_GPR_).

Statistical analyses, regressions, SMB reconstructions, visualizations, and other statistical analyses were performed using the R programming language with packages *ggplot2*, *RColorBrewer*, *gridExtra*, *cowplot*, and *tidyverse* and with Adobe Illustrator. QGIS was used for spatial analyses and map creation using data produced here or cited in image captions.

## Supplementary information

Supplementary Information

Peer Review File

## Data Availability

The data generated in this study have been deposited in the PANGAEA online repository^73,74^ at 10.1594/PANGAEA.941480 and 10.1594/PANGAEA.941491. All original source and figure data are available in this data or produced using the code linked below.

## Code availability

Code for reproducing analyses and figure creation^75^ is available at 10.5281/zenodo.6806404.
