# Supplementary material for: Sunlight-driven nitrate loss records Antarctic surface mass balance
Source: Nat Commun. 2022 Jul 25;13:4274. doi: 10.1038/s41467-022-31855-7 (PMC9314437; doi:10.1038/s41467-022-31855-7)
Supplement: Supplementary file 1 — Supplementary Information [file 41467_2022_31855_MOESM1_ESM.pdf]

Sunlight-driven nitrate loss records Antarctic surface mass balance

Supplementary Information

Supplementary Discussion 1. Theoretical framework modeling

Linear regressions of the relationship in Eq. (15) were modeled for the field database sites by parameterizing factors other than SMB based on best known estimates. Values for I_0 were calculated as spectral actinic flux in five bins over the wavelength range 290–350 nm using the online TUV calculator[1,2], and calculated for each site using specific latitude, longitude, and elevation, and assuming clear skies, baseline aerosols, and a total ozone column (TCO) of 300 Dobson units (DU). Mean annual I_0 values were determined for each bin by multiplying the maximum daily mean I_0 (i.e., on the summer solstice) by a factor that compensates for different durations of polar night based on latitude ($I_{0.mean.yr} = I_{0.max.day} \times (0.00780\ |lat.deg| + 1.02)$). A σI_0 value was calculated for each database site by multiplying wavelength-appropriate absorption cross section values from Chu and Anastasio, 2003[3], to the mean annual I_0 bins and summing across all wavelengths.

For the other parameters, a single "current best-supported" estimate was chosen to calculate a base set of predicted $\delta^{15}N_{NO3arc}$ values using the database SMBs and calculated σI_0 values, while four additional estimates were chosen for each parameter to cover a more complete range of possible values as reported in literature (Supplementary Table 1). For each parameter, regressions of predicted $\delta^{15}N_{NO3arc}$ vs. SMB^{-1} were calculated over the range of parameter values by holding all other parameters to their base set values. Minimum and maximum possible regressions were also calculated by combining all lowest or all highest parameter estimates.

Isotopic fractionation factors and *e*-folding depths cover the range reported from field observations[4–7,11,13,14]. The highest reported values for ϕ (0.05–0.6)[8,18] were not used in our model, as they produce extremely high $\delta^{15}N_{NO3arc}$ values (600–3000 ‰) that are well above the highest reported values for $\delta^{15}N_{NO3}$[4,5]. We examined the effect of different TCO values by changing the TCO parameter in the online TUV calculator to produce different I_0 values than the base state. The chosen TCO values represent general seasonal minimums and maximums (150, 400 DU) observed at Halley Station[15], as well as the mean TCO during the light season (Oct–Mar) both before stratospheric ozone destruction (1956–1979: 320 DU) and after (1990–2015: 240 DU).

Finally, it is important to recognized that the $\delta^{15}N_{NO3_0}$ value in our model incorporates the $\delta^{15}N_{NO3}$ values of both newly deposited NO_3^- (i.e., with a low $\delta^{15}N_{NO3}$ that matches typical atmospheric NO_3^-) and recycled NO_3^- that is deposited from the re-oxidation of recently photolyzed NO_3^- and has a higher $\delta^{15}N_{NO3}$ value. Because dry interior sites have greater NO_3^- recycling[10,11], their $\delta^{15}N_{NO3_0}$ values will be higher than observed at more coastal and wetter sites. In addition to a constant atmospheric value (−20 ‰), we have parameterized the spatial variability in $\delta^{15}N_{NO3_0}$ values through linear regression of reported skin layer NO_3^- values vs. SMB^{-1} along four East Antarctic transects: DDU–DC+EAIIST (r^2 = 0.44)[4], DDU–DC (r^2 =

0.66)[4], Zhongshan–DA, minus 400–800 km section (r^2 = 0.53)[17], and Zhongshan–DA, smoothing-simplified (r^2 = 0.86)[17].

Using the SMB values in our dataset and current best-supported parameter estimates (Supplementary Table 1) for the other variables and constants in Eq. (15), we predict a modeled regression where $\ln(\delta^{15}N_{NO3arc} + 1) = 3.81\ SMB^{-1} - 0.02$ (Supplementary Fig. 1). Although the slope is different from the field data regression ($\ln(\delta^{15}N_{NO3arc} + 1) = 6.98\ SMB^{-1} - 0.02$), sensitivity testing across different parameter estimates reveals that modeled slope values vary widely from 0.01 to 18.4 when the full combined possibilities of parameter values are considered, and only small adjustments to parameter values within reported variability produce model regressions that closely match the field regression (e.g., $\ln(\delta^{15}N_{NO3arc} + 1) = 6.96\ SMB^{-1} - 0.00$ when $\phi = 0.42$ and other parameters are held at best estimates). While it is difficult to determine which parameter value combination is the most representative of actual conditions, 42% of the total modeled slope range is due to different reported ϕ values alone, with lesser contributions from uncertainty in $\delta^{15}N_{NO3_0}$, z_e, and a. As proposed values for ϕ widely disagree across the literature, a more precise estimate of ϕ is needed before we can robustly judge values of other parameters. Despite this, we are fairly confident that changes in total column ozone concentration (TCO) due to stratospheric ozone destruction should only exert a minor influence on $\delta^{15}N_{NO3arc}$ values, as $\delta^{15}N_{NO3arc}$ values modeled with modern TCO are just 13 ‰ higher than pre-1970s TCO[15] at the most sensitive (i.e., driest) sites (Supplementary Fig. 1d).

Additional refinements to theoretical model parameters may improve the performance of $\delta^{15}N_{NO3arc}$ as a proxy for SMB. Because the parameters with known and defined spatial variability (i.e., I_0, TCO, $\delta^{15}N_{NO3_0}$) linearly covary with SMB^{-1}, our model sensitivity testing did not identify any additional spatial correction factors that improved the $SMB_{\delta15N}$ predictions over the simple $\delta^{15}N_{NO3arc}$ vs. SMB^{-1} linear regression. However, while we assumed z_e to be constant across all sites due to very limited field observations[11,13,14], we acknowledge that z_e values likely vary spatially in some manner with changes in snow grain size, glazed surface presence, and impurity content. Quantifying this spatial variability through increased field observations and/or remote sensing will enable more accurate modeling of z_e values that could potentially reduce some of the $SMB_{\delta15N}$ regression residuals.

Similarly, $\delta^{15}N_{NO3_0}$ (i.e., the $\delta^{15}N_{NO3}$ ≈1 cm below the surface) clearly exhibits spatial variability due to the effect of NO_3^- recycling on local NO_3^- supply[4,17], and we parameterized $\delta^{15}N_{NO3_0}$ through linear regressions of reported surface layer $\delta^{15}N_{NO3}$ and SMB^{-1} along Antarctic transects. However, the exact depth of a surface layer sample often varies between different campaigns and sampling personnel, and a sample that is 1–2 cm deep may have a substantially higher $\delta^{15}N_{NO3}$ value than a sample that only collects the loose snow above the surface crust. Thus, intercomparing datasets is difficult despite a relatively expansive set of $\delta^{15}N_{NO3}$ values from surface snow[4,5,17], and the true spatial distribution of $\delta^{15}N_{NO3_0}$ is ill-defined. For future sampling, we recommend that researchers separate samples of the loose, uppermost surface snow from the 0-2 cm depth sample and take multiple samples per site when possible. Using a more standardized sampling approach, we can better quantify this

important parameter while also learning more about the initial sourcing and deposition of Antarctic NO_3^-.

Finally, the $SMB_{\delta 15N}$ proxy assumes that the non-SMB parameters are not changing over the period of time covered by the SMB reconstruction. Holding the parameters of quantum yield and fractionation constant, the values modeled in our sensitivity tests represents not presumed natural variability over time or space, but rather uncertainty in knowing the true value of these constants. The other factors of TCO, initial $\delta^{15}N_{NO3}$, and *e*-folding depth have a greater risk of varying enough in the past to adversely affect $SMB_{\delta 15N}$ reconstructions, particularly if the ice core extends back into glacial periods when the Antarctic and global environments were drastically different. The limited sensitivity of $\delta^{15}N_{NO3}$ to modern ozone variability (Supplementary Fig. 1d) suggests that impacts from past ozone concentration difference will be limited unless extreme changes occurred. Atmospheric $\delta^{15}N_{NO3}$ values are not likely to have ranged far outside of the −20 – +20 ‰ range observed based on the set of known atmospheric oxidation reactions[19,20], and the stability of atmospheric $\delta^{15}N_{NO3}$ values over millennial timescales could be analyzed through $\delta^{15}N_{NO3arc}$ values from a deep ice core drilled from a high SMB site where atmospheric values are largely preserved due to little photolytic loss. Changes in *e*-folding depth in the past are difficult to consider as the spatial and temporal variability in *e*-folding depth is poorly known even for today. Further study into modern distributions and controls on *e*-folding depth is needed to better constrain this variable and consider its potential changes in the past.

Supplementary Discussion 2. Effect of SMB choice on linear regressions

To investigate if the choice of SMB source (SMB_{ground} vs. SMB_{adjMAR}) introduced any significant differences in linear regression coefficient estimates compared to the primary dataset that used the best available SMB for each site, we calculated regressions for Eq. (1) with three additional dataset variants: 1) all database sites, but using SMB_{adjMAR} for all sites, 2) only sites with SMB_{ground} data, and 3) only sites without SMB_{ground} data (i.e., sites that only have SMB_{adjMAR}) (Supplementary Table 2, Supplementary Fig. 2). Paired t-tests of regression coefficients between the different dataset variants revealed that differences in coefficient values were not statistically different from zero (p-values: 0.27–0.99). This suggests that the modeled SMB_{adjMAR} values are indeed similar enough to the field-observed SMB_{ground} values that they can be interchanged and merged without substantial changes to the resulting linear relationships.

Using Eq. (1), we observe that the small differences in the SMB variants' regressions do not propagate into substantial differences when predicting $\delta^{15}N_{NO3arc}$ for a given SMB (Supplementary Table 3). Notably, the differences between the $\delta^{15}N_{NO3arc}$ values predicted at a given SMB for the different variant regressions are small compared to the magnitude of $\delta^{15}N_{NO3arc}$ changes across the potential range of SMBs. The similarities in regression coefficients, relationship strength, and $\delta^{15}N_{NO3arc}$ predictions across the different SMB dataset variants suggests that while SMB_{ground} observations should be favored as the highest quality data due to their onsite origin, model-based SMBs can be incorporated without adversely affecting results. However, this incorporation must compensate for any identified bias and

offset between modeled SMBs and real-world observations, as performed in our SMB_{adjMAR} corrections. Continued improvements to Antarctic regional climate model accuracy will increase the value of model SMB estimates in future examinations of the relationship between $\delta^{15}N_{NO3arc}$ and SMB.

Supplementary Discussion 3. Post-depositional changes to $\delta^{15}N_{NO3}$ beneath the photic zone at dry sites

The current framework for NO_3^- dynamics in Antarctic snow holds that post-depositional changes that affect $\delta^{15}N_{NO3}$ values are limited the photic zone. Once NO_3^- is buried beneath the lower bound of the photic zone (i.e., the archived zone), its $\delta^{15}N_{NO3}$ value is stable and preserved indefinitely. However, this assumption is apparently violated at very dry sites (i.e., dome summits or the remote interior plateau where SMB < 40 kg m^{-2} a^{-1}) as $\delta^{15}N_{NO3arc}$ values deeper than 2 m can be substantially lower than those near 1 m depth (i.e., the bottom of the photic zone). At Dome C, for example, five 1 m depth samples taken in three separate years (2004, 2007, 2010) have values between 222 ‰ and 302 ‰, and other pit samples taken in the ~100 km radius of Dome C and at other Antarctic dome summits have similarly high values. In contrast, a deeper ice core sampled between 6.75 and 72.33 m at Dome C (core *DC14*, not currently published) has a mean $\delta^{15}N_{NO3arc}$ value of only 170 ‰ and no single sample (0.33 m resolution) is greater than 242 ‰.

This major drop in $\delta^{15}N_{NO3}$ value below the bottom of the photic zone is observed at other ultra-dry sites as well. The mean $\delta^{15}N_{NO3arc}$ value for a core taken from Dome A[21] is 90 ‰ lower than the mean $\delta^{15}N_{NO3arc}$ from 0.3 to 3.0 m in a Dome A pit (264 ‰ vs. 354 ‰)[5], and Holocene-age samples reported from a Vostok core[22] have a mean $\delta^{15}N_{NO3arc}$ value of 153 ‰ compared to local pit $\delta^{15}N_{NO3arc}$ values of 259–335 ‰. Finally, for two deep snow pits, P7 at Dome A[5] and DC04 at Dome C[16], the highest $\delta^{15}N_{NO3arc}$ values are found just below the photic zone boundary, and then $\delta^{15}N_{NO3arc}$ values decline with depth by ~100 ‰ over the next 1–3 meters. No such large discrepancy between basal photic zone values and deeper archived zone values has been observed at ABN or at sites sampled outside of the ultra-dry dome summits and high interior plateau.

The root cause for these $\delta^{15}N_{NO3}$ observations is not known, and further investigation is hampered by limited available observations and data. Still, the differences are too extreme to be attributed to recent SMB changes, and the $\delta^{15}N_{NO3}$ peaks often do not match well in timing with each other or with the timeline of stratospheric ozone destruction above Antarctica in the late 20th century[23]. Additionally, our modeling suggests that this decline in annual TCO would only increase $\delta^{15}N_{NO3arc}$ values by ≈13 ‰ at ultra-dry sites. Notably, the ABN core has no major difference in $\delta^{15}N_{NO3}$ values between the periods 1980–2000 CE and 1960–1980 CE, arguing that this phenomenon is indeed limited to ultra-dry sites and not a result of spatially broad effects from the diminished ozone column over Antarctica.

We propose that the sub-photic zone transport and re-oxidation of photolytic NO_x through wind pumping and other convective drivers[24–26] may supply NO_3^- with low $\delta^{15}N_{NO3}$ to depths below 1 m. This effect would be enhanced at the driest sites because firn convection zones are deeper at drier sites[25,26], the very low NO_3^- concentrations (~10-50 ng g^{-1}) in the archived

zone at drier sites are sensitive to even small additions of re-oxidized NO_3^-, and slow burial rates keep NO_3^- longer in the zone where interstitial firn air can exchange. Clearly, more observations of $\delta^{15}N_{NO3}$ profiles and field observations in the uppermost archived zone are needed to fully understand NO_3^- dynamics at ultra-dry sites, and to potentially expand the $SMB_{\delta 15N}$ proxy to these sites as well.

Supplementary Discussion 4. Reconstructing SMB at WAIS Divide and Dome A

Very few other ice cores in Antarctica have reported $\delta^{15}N_{NO3arc}$ data, and those that have $\delta^{15}N_{NO3arc}$ data are from sites whose modern SMB values are outside the range of 40–200 kg m^{-2} a^{-1} best suited for our $SMB_{\delta 15N}$ proxy. Still, examining the results of the $SMB_{\delta 15N}$ proxy at its environmental extremes can still provide important context on the applicability and possible future expansion of $SMB_{\delta 15N}$ methods. We here apply our SMBd15N proxy to $\delta^{15}N_{NO3arc}$ data from two other ice cores, WD06A from the West Antarctic Ice Sheet (WAIS) Divide[27] and DA2005[21,28] from Dome A, that are respectively wetter (220 kg m^{-2} a^{-1})[29] and drier (23 kg m^{-2} a^{-1})[30] than the ideal range for the $SMB_{\delta 15N}$ proxy (Supplementary Fig. 3).

For WAIS Divide (Supplementary Fig. 3a), our $SMB_{\delta 15N}$ reconstruction differs by −10 – +93 kg m^{-2} a^{-1} compared to the SMB values calculated from annual layer thickness corrected for ice strain flow and densification (equivalent to the $SMB_{density}$ method described in the main text). While this ≈+45 kg m^{-2} a^{-1} offset appears rather large, the general flatness of the relationship between $\delta^{15}N_{NO3arc}$ and SMB where SMB > 200 kg m^{-2} a^{-1} means that this offset is the equivalent of the $\delta^{15}N_{NO3arc}$ values being only ≈6 ‰ lower than expected based on Eq. (1). Despite the offset, the patterns of variation are similar between the two SMB time series ($r = 0.61$, $p \ll 0.001$, $n = 34$), with both showing a sustained SMB decrease between 1500 and 500 yr BP. This suggests that the $\delta^{15}N_{NO3arc}$ value in high accumulation Antarctic sites is still primarily reflecting changes in local SMB despite the relatively limited photolytic NO_3^- loss. Interestingly, calculating $SMB_{\delta 15N}$ values using the regression determined only from sites with ground-observed SMB values (Supplementary Table 2) results in a much better agreement between $SMB_{\delta 15N}$ and $SMB_{density}$. This "ground-observations only" SMB regression does not accurately reconstruct SMBs at the ABN or Dome A sites, but its excellent performance at WAIS suggests that it may be possible to expand the $SMB_{\delta 15N}$ proxy to sites wetter than our cautious 200 kg m^{-2} a^{-1} limit if additional sampling of $\delta^{15}N_{NO3arc}$ values at sites with well-constrained SMB values can further refine and improve the overall regression for Eq. (1).

For Dome A (Supplementary Fig. 3b), $SMB_{\delta 15N}$ values are 3.2–7.6 kg m^{-2} a^{-1} higher than $SMB_{density}$ values, and there is no observed significant correlation ($r = -0.21$, $p = 0.39$, $n = 17$). While the offsets in SMB values are relatively small in magnitude, they are well outside than the known SMB maxima observed for Dome A and a substantial region surrounding it. Additionally, because Dome A is an ultra-dry site that falls where the relationship between $\delta^{15}N_{NO3arc}$ and SMB is nearly vertical (Figure 2b), this small offset actually reflects that the $\delta^{15}N_{NO3arc}$ values at Dome A are 50–75 ‰ lower than what our regression expects for the observed $SMB_{density}$. This difference follows the observations reported for all other ultra-dry

sites in Supplementary Discussion 3, possibly because NO_3^- with low $\delta^{15}N_{NO3}$ values is being transported below the photic zone through ventilation.

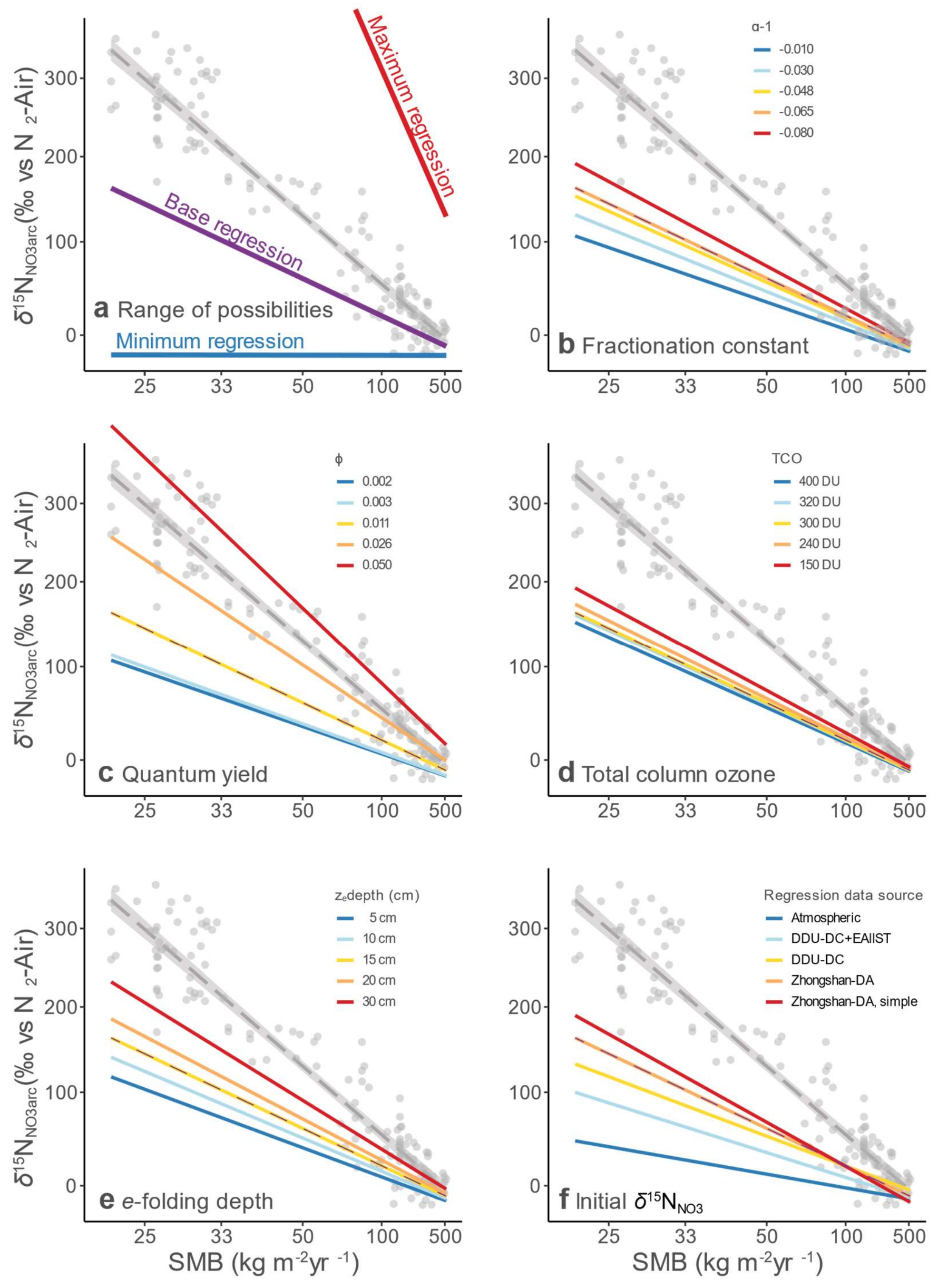

Supplementary Figure 1. Sensitivity testing results for parameters in the SMB vs. $\delta^{15}N_{NO3arc}$ relationship. Regressions for ln($\delta^{15}N_{NO3arc}$ + 1) vs. SMB^{-1} at database sites where $\delta^{15}N_{NO3arc}$ values are modeled through Eq. (15) with parameter values in Supplementary Table 1. The actual field samples and their ln($\delta^{15}N_{NO3arc}$ + 1) vs. SMB^{-1} regression from Fig. 2c are shown as gray circles and lines, respectively. The base model regression, calculated with the best-supported parameter estimates, is shown in purple in **a**, which the extreme models of all parameter combinations are shown as the minimum (blue) and maximum (red) model regressions. The other plots show the sensitivity of the model to changes in individual parameter values of **b** $\delta^{15}N_{NO3}$ isotopic fractionation constant, **c** quantum yield of NO_3^- photolysis, **d** total column ozone, **e** *e*-folding depth, and **f** initial $\delta^{15}N_{NO3}$ value, with the value used in the base model value indicated with purple dashes in each parameter's plot.

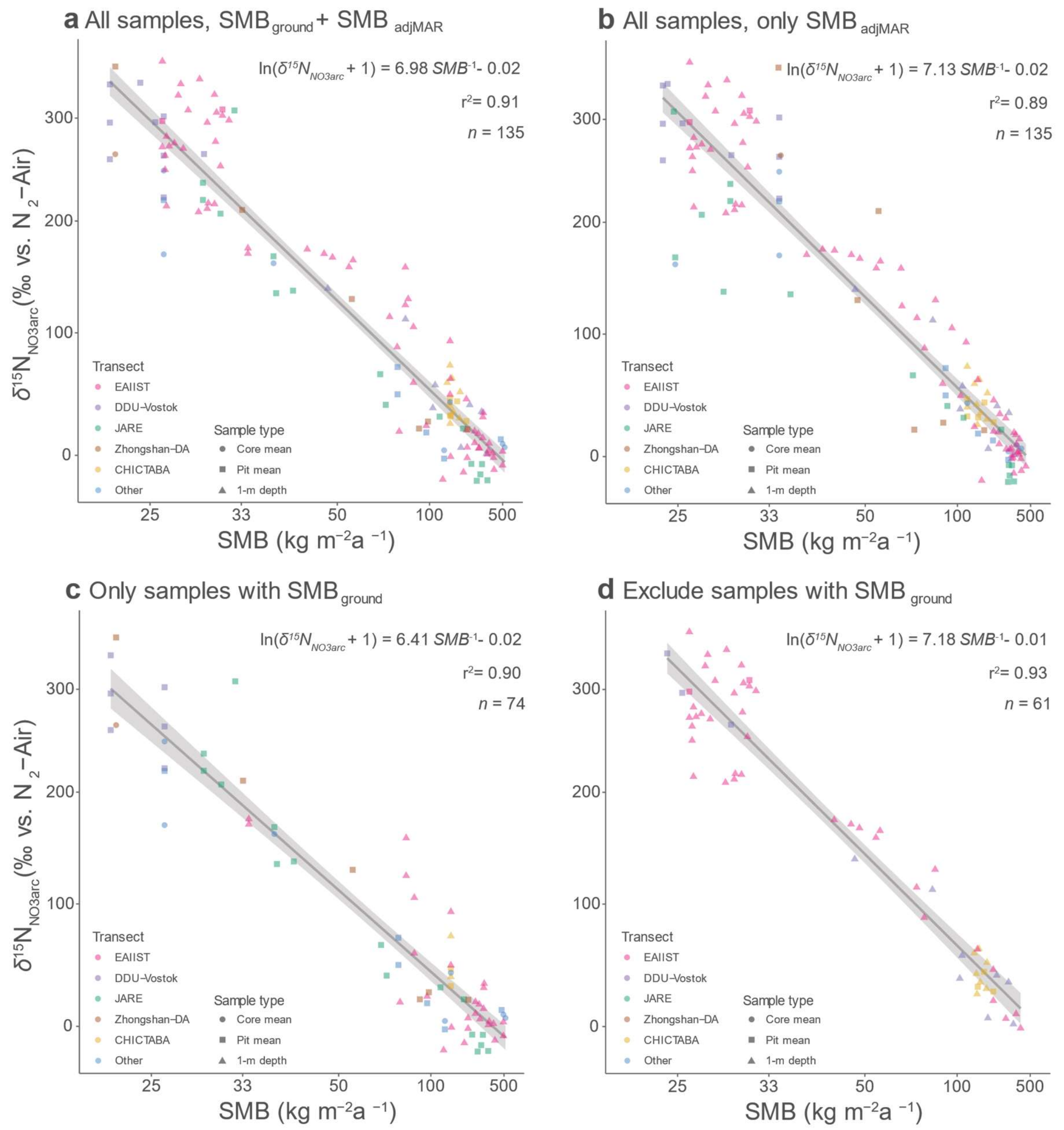

Supplementary Figure 2. Comparison of regression results for different SMB dataset variants. Scatter plots and linear regressions of Eq. (1) with different variants of the SMB dataset (Supplementary Table 2) plotted separately (**a–d**). Linear regressions (gray solid lines) are shown with the 95% confidence intervals of the regression shaded.

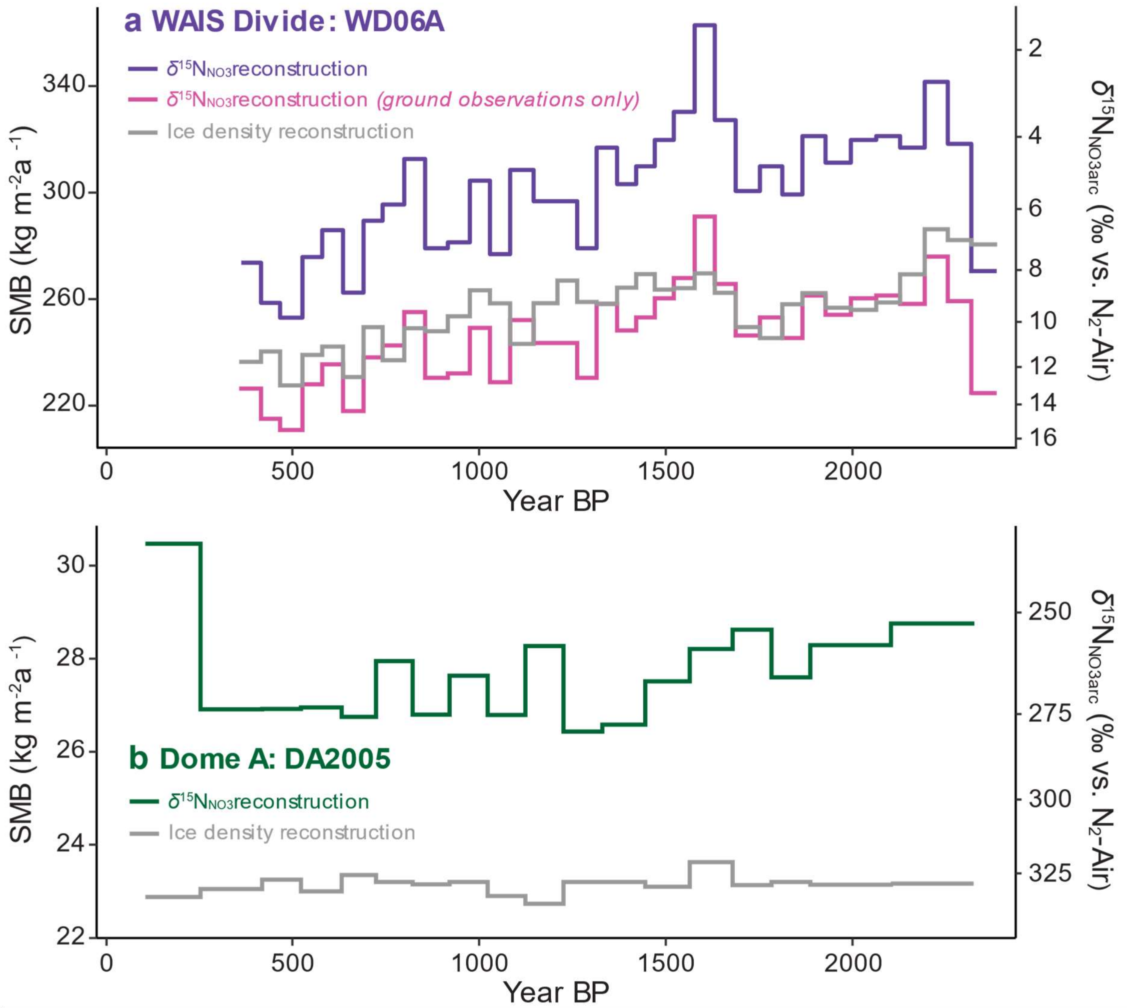

Supplementary Figure 3. Application of the $SMB_{\delta 15N}$ proxy to other available sites. Comparison of SMB reconstructions from $\delta^{15}N_{NO3arc}$ (colored lines) and ice density (gray lines) for the **a** WD06A[27] and **b** DA2005[21,28] ice cores. For WAIS, $SMB_{\delta 15N}$ values were calculated both using the regression parameters described in the main text (purple line) and with the regression parameters determined using only sites with ground-observed SMB values (pink line). The $\delta^{15}N_{NO3arc}$ y-axis at right is the conversion of the SMB y-axis to $\delta^{15}N_{NO3arc}$ using Eq. (1), and represent the actual $\delta^{15}N_{NO3arc}$ values observed in the ice cores through the $SMB_{\delta 15N}$ proxy (purple and green lines) and the expected $\delta^{15}N_{NO3arc}$ values for the given $SMB_{density}$ observations based on the Eq. (1) relationship.

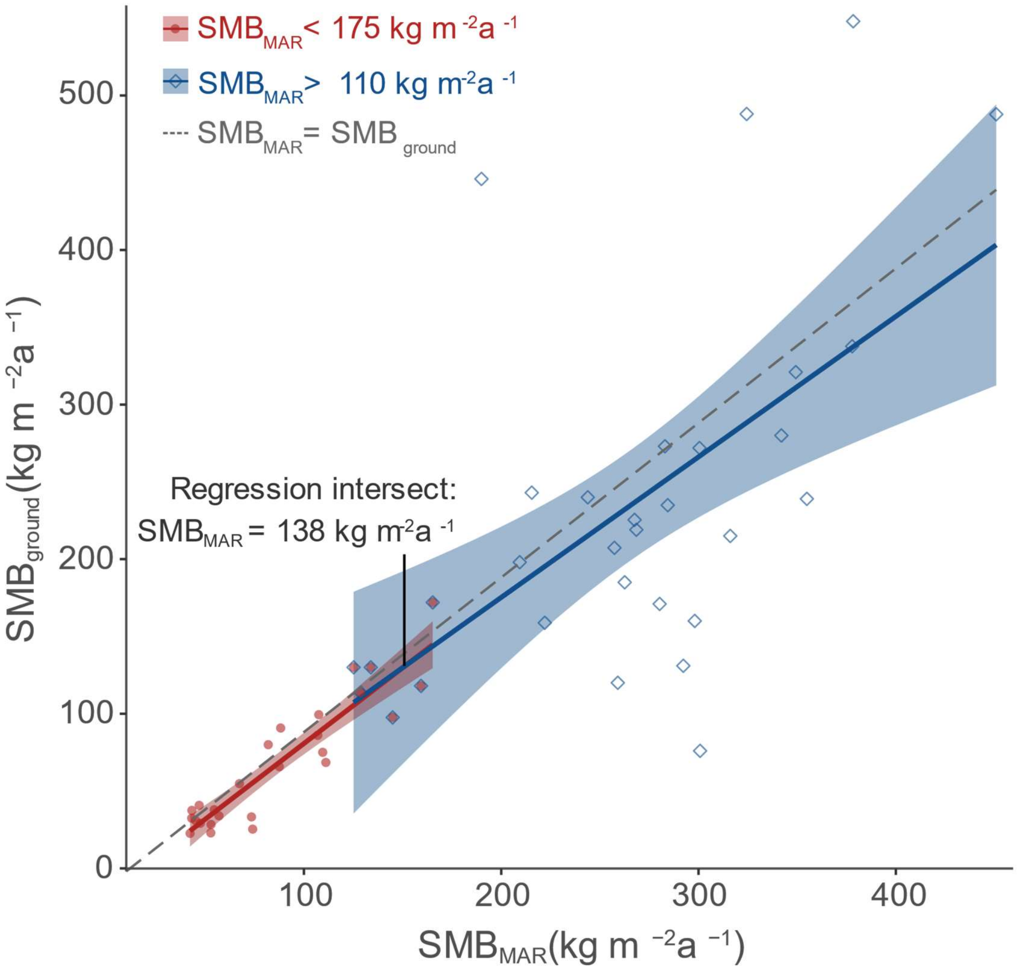

Supplementary Figure 4. Comparison of ground-observed SMB with MAR output SMB. Linear regressions of SMB_{ground} versus SMB_{MAR} for the period 1979–2017 at the 51 sites with SMB_{ground} observations, with 95% confidence intervals of the regressions shaded. Sites are subset for two overlapping regressions that intersect at (138, 130). These linear regressions were applied to the SMB_{MAR} values for all sampling sites to produce the SMB_{adjMAR} used in analyses. The dashed line represents a slope of 1 (i.e., if the SMB_{MAR} perfectly matched the SMB_{ground}).

Supplementary Table 1. Parameter values used to model $\delta^{15}N_{NO3arc}$ values through Eq. (1). The values used in the base model (i.e., those assumed to be current best-supported estimates) are bolded.

Parameter	Parameter Values Used in Modeling				
$(a\text{-}1)$	−10 ‰[4,5]	−30 ‰[4,5]	−48 ‰[6]	**−56 ‰**[7]	−80 ‰[4,5]
ϕ	0.002[3]	0.003[3,8]	**0.011**[9]	0.026[10]	0.050[8]
z_e	5[11,12]	10[13,14]	**15**[13,14]	20[13,14]	30[13]
TCO	150[15]	240[15]	**300**[15]	320[15]	400[15]
$\delta^{15}N_{NO30}$	−20 ‰ 4,16	$\frac{1217.5}{SMB} - 21.4$ ‰ (4 + EAIIST)	$\frac{1830.2}{SMB} - 15.5$ ‰ 4	$\mathbf{\frac{2730.5}{SMB} - 24.0}$ **‰** 17	$\frac{3554.4}{SMB} - 32.4$ ‰ 17

Supplementary Table 2. Linear regressions of different SMB dataset variants. Slope units are in m^2 a kg^{-1}.

			Eq. 1 $\ln(\delta^{15}N_{NO3arc} + 1) = \frac{a}{SMB} + b$		
**Samples**	**SMB Data**	***n***	**Slope (*a*)**	**Intercept (*b*)**	***r***
All samples	Ground + Adj.-MAR	135	6.98 ± 0.19	-0.02 ± 0.01	0.91
All samples	Adj.-MAR	135	7.13 ± 0.22	-0.02 ± 0.01	0.89
Samples with SMB_{ground}	Ground	74	6.41 ± 0.25	-0.02 ± 0.01	0.90
Samples without SMB_{ground}	Adj.-MAR	61	7.18 ± 0.26	-0.01 ± 0.01	0.93

Supplementary Table 3. Predicting $\delta^{15}N_{NO3arc}$ values for given SMB values. Values of $\delta^{15}N_{NO3arc}$ calculated for a given SMB based on the SMB vs. $\delta^{15}N_{NO3arc}$ linear regression (Eq. (1)). Results provided based on all four linear regressions (Supplementary Table 2).

**Samples**	**SMB Data**	**$\delta^{15}N_{NO3o}$ (‰)**	**$\delta^{15}N_{NO3arc}$ (‰) when SMB ($kg\ m^{-2}\ a^{-1}$) =**					
			500	*200*	*100*	*50*	*20*	*10*
All samples	Ground + Adj.-MAR	-17.6	-3.8	17.3	53.5	129.6	392.9	974.8
All samples	Adj.-MAR	-16.3	-2.2	19.4	56.4	134.4	405.0	1006.8
Samples with SMB_{ground}	Ground	-20.3	-7.7	11.6	44.5	113.6	349.5	859.0
Samples without SMB_{ground}	Adj.-MAR	-7.8	6.5	28.4	66.0	145.4	420.7	1034.4

Supplementary Table 4. Corrective regressions for JARE data. Linear regressions of $\delta^{15}N_{NO3arc}$ vs. $\delta^{15}N_{NO3.X}$ (where X is 30, 50, or 80 cm) calculated from non-JARE pit data in the $\delta^{15}N_{NO3}$ database.

Depth correction	Slope (‰ / ‰)	Intercept (‰)	r^2
0–30 cm	1.9±0.1	-2.4±11.3	0.89
0–50 cm	1.6±0.1	-1.7±8.2	0.94
0–80 cm	1.5±0.1	-0.9±7.8	0.94

Supplementary Table 5. Comparison of pre- and post-correction JARE data. The $\delta^{15}N_{NO3}$ values for JARE sites included in our database as originally reported by Noro et al. (2018) and the $\delta^{15}N_{NO3arc}$ values corrected here to account for photic zone snow included in the original samples. Samples with original $\delta^{15}N_{NO3}$ values < 0 ‰ (italicized) were not corrected.

JARE site	Depth (cm)	Original $\delta^{15}N_{NO3}$ (‰)	Corrected $\delta^{15}N_{NO3arc}$ (‰)
Z2	0–80	20.6	30.8
IM0	0–50	25.7	40.3
NMD304	0–50	41.1	65.4
MD590	0–50	83.5	134.7
DF1	0–30	127.3	236.5
NDF	0–30	111.7	207.2
Plateau S	0–30	165.5	308.1
S80	0–30	90.7	167.8
Fuji Pass	0–30	74.3	137.0
DF2	0–30	118.6	220.1
S30	*0–50*	*-19.0*	*-19.0*
H42	*0–50*	*-6.6*	*-6.6*
H68	*0–50*	*-14.5*	*-14.5*
H88	*0–50*	*-19.4*	*-19.4*
H108	*0–50*	*-6.4*	*-6.4*
H128	0–50	14.1	21.3

Supplementary Table 6. Sites and SMB data used to bias-correct the original MAR output. A list of all sampling sites that have a SMB_{ground} observation with corresponding values of original SMB_{MAR} and SMB_{adjMAR} (Supplementary Fig. 4). The difference between the SMB_{adjMAR} and SMB_{MAR} values is given in the second to last column with the ratio between SMB_{adjMAR} and SMB_{MAR} in the final column.

Site	SMB_{ground} (kg m^{-2} a^{-1})	SMB_{MAR} (kg m^{-2} a^{-1})	SMB_{adjMAR} (kg m^{-2} a^{-1})	SMB_{adjMAR} – SMB_{MAR} (kg m^{-2} a^{-1})	SMB_{adjMAR} / SMB_{MAR}
Vostok	22.6	30.4	24.1	-6.3	0.79
DomeA	22.9	40.9	34.4	-6.5	0.84
ZtoA-P6	25.4	62.1	55.2	-6.9	0.89
DomeC	28.4	41.0	34.5	-6.5	0.84
DomeF	29.2	35.6	29.2	-6.4	0.82
NDF	30.9	33.2	26.8	-6.4	0.81
Plateau S	32.4	31.2	24.8	-6.4	0.79
ZtoA-P5	33.3	61.5	54.6	-6.9	0.89
preeaiist.18	34.0	47.8	41.1	-6.7	0.86
S80Jare	37.5	31.3	24.9	-6.4	0.80
MD590	37.9	42.6	36.0	-6.6	0.85
Fuji Pass	40.7	35.0	28.6	-6.4	0.82
ZtoA-P4	54.8	55.4	48.6	-6.8	0.88
NMD304	65.8	75.6	68.4	-7.2	0.90
IM0	68.5	99.2	91.6	-7.6	0.92
Kohnen	75.0	97.6	90.0	-7.6	0.92
posteaiist.asuma05	76.0	288.9	266.9	-22.0	0.92
preeaiist.15	80.0	70.0	62.9	-7.1	0.90
preeaiist.13	86.0	95.1	87.5	-7.6	0.92
ZtoA-P3	90.7	76.3	69.1	-7.2	0.91
CPH.D5	97.6	139.7	124.8	-8.3	0.89
ZtoA-P2	99.4	95.6	88.0	-7.6	0.92
Z2	113.5	116.9	108.9	-8.0	0.93
posteaiist.stop36	118.0	147.5	138.3	-9.2	0.94
CPH.D24	120.0	247.2	228.9	-18.3	0.93
preeaiist.12	130.0	113.3	105.4	-7.9	0.93

ABN	130.0	122.0	113.9	-8.1	0.93
posteaiist.asuma06	131.0	280.4	259.1	-21.3	0.92
H128	158.8	210.2	195.3	-14.9	0.93
preeaiist.06	160.0	286.2	264.4	-21.8	0.92
preeaiist.07	171.0	269.5	249.2	-20.3	0.92
ZtoA-P1	172.0	153.4	143.7	-9.7	0.94
H108	185.0	250.7	232.1	-18.6	0.93
preeaiist.09	198.0	201.0	186.9	-14.1	0.93
H88	207.3	245.5	227.4	-18.1	0.93
posteaiist.asuma04	215.0	304.2	280.8	-23.4	0.92
posteaiist.asuma09	219.0	256.6	237.5	-19.1	0.93
H68	225.4	255.7	236.7	-19.0	0.93
H42	234.9	272.5	251.9	-20.6	0.92
posteaiist.asuma02	239.0	343.0	316.0	-27.0	0.92
posteaiist.asuma10	240.0	232.0	215.1	-16.9	0.93
posteaiist.asuma11	243.0	203.6	189.3	-14.3	0.93
S30-JARE	271.9	288.6	266.6	-22.0	0.92
posteaiist.asuma07	273.0	271.1	250.7	-20.4	0.92
preeaiist.04	280.0	330.1	304.3	-25.8	0.92
posteaiist.asuma01	321.0	337.4	310.9	-26.5	0.92
preeaiist.03	337.7	366.0	337.0	-29.0	0.92
cph.d17	446.0	178.1	166.1	-12.0	0.93
preeaiist.02	487.8	439.0	403.3	-35.7	0.92
asuma.2016.2	488.0	312.5	288.3	-24.2	0.92
asuma.2016.1	548.0	366.5	337.4	-29.1	0.92

SUPPLEMENTARY REFERENCES

1. NCAR UCAR. Online TUV Calculator, v5.3. https://www.acom.ucar.edu/Models/TUV/Interactive_TUV/ (2021).
2. Madronich, S. & Flocke, S. The role of solar radiation in atmospheric chemistry. in *Handbook of Environmental Geochemistry* 1–26 (Springer-Verlag, 1998).
3. Chu, L. & Anastasio, C. Quantum yields of hydroxyl radical and nitrogen dioxide from the photolysis of nitrate on ice. *J. Phys. Chem. A* **107**, 9594–9602 (2003).
4. Erbland, J. *et al.* Air-snow transfer of nitrate on the East Antarctic Plateau - Part 1: Isotopic evidence for a photolytically driven dynamic equilibrium in summer. *Atmospheric Chemistry and Physics* **13**, 6403–6419 (2013).
5. Shi, G. *et al.* Investigation of post-depositional processing of nitrate in East Antarctic snow: isotopic constraints on photolytic loss, re-oxidation, and source inputs. *Atmospheric Chemistry and Physics* **15**, 9435–9453 (2015).
6. Berhanu, T. *et al.* Laboratory study of nitrate photolysis in Antarctic snow. II. Isotopic effects and wavelength dependence. *Journal of Chemical Physics* **140**, (2014).
7. Berhanu, T. *et al.* Isotopic effects of nitrate photochemistry in snow: a field study at Dome C, Antarctica. *Atmospheric Chemistry and Physics* **15**, 11243–11256 (2015).
8. Meusinger, C., Berhanu, T. A., Erbland, J., Savarino, J. & Johnson, M. S. Laboratory study of nitrate photolysis in Antarctic snow. I. Observed quantum yield, domain of photolysis, and secondary chemistry. *The Journal of Chemical Physics* **140**, 244305 (2014).
9. Benedict, K. B., McFall, A. S. & Anastasio, C. Quantum yield of nitrite from the photolysis of aqueous nitrate above 300 nm. *Environ. Sci. Technol.* **51**, 4387–4395 (2017).
10. Erbland, J. *et al.* Air-snow transfer of nitrate on the East Antarctic Plateau - Part 2: An isotopic model for the interpretation of deep ice-core records. *Atmospheric Chemistry and Physics* **15**, 12079–12113 (2015).
11. Winton, V. H. L. *et al.* Deposition, recycling, and archival of nitrate stable isotopes between the air–snow interface: comparison between Dronning Maud Land and Dome C, Antarctica. *Atmos. Chem. Phys.* **20**, 5861–5885 (2020).
12. Wolff, E., Jones, A., Martin, T. & Grenfell, T. Modelling photochemical NOX production and nitrate loss in the upper snowpack of Antarctica. *Geophysical Research Letters* **29**, (2002).
13. Zatko, M. C. *et al.* The influence of snow grain size and impurities on the vertical profiles of actinic flux and associated NOx emissions on the Antarctic and Greenland ice sheets. *Atmos. Chem. Phys.* **13**, 3547–3567 (2013).
14. France, J. L. *et al.* Snow optical properties at Dome C (Concordia), Antarctica; implications for snow emissions and snow chemistry of reactive nitrogen. *Atmos. Chem. Phys.* **11**, 9787–9801 (2011).
15. Shanklin, J. BAS Antarctic ozone legacy data site. https://legacy.bas.ac.uk/met/jds/ozone/index.html#data (2021).
16. Frey, M., Savarino, J., Morin, S., Erbland, J. & Martins, J. Photolysis imprint in the nitrate stable isotope signal in snow and atmosphere of East Antarctica and implications for reactive nitrogen cycling. *Atmospheric Chemistry and Physics* **9**, 8681–8696 (2009).

17. Shi, G. *et al.* Nitrate deposition and preservation in the snowpack along a traverse from coast to the ice sheet summit (Dome A) in East Antarctica. *The Cryosphere* **12**, 1177–1194 (2018).
18. Zhu, C., Xiang, B., Chu, L. T. & Zhu, L. 308 nm photolysis of nitric acid in the gas phase, on aluminum surfaces, and on ice films. *J. Phys. Chem. A* **114**, 2561–2568 (2010).
19. Savarino, J., Kaiser, J., Morin, S., Sigman, D. & Thiemens, M. Nitrogen and oxygen isotopic constraints on the origin of atmospheric nitrate in coastal Antarctica. *Atmospheric Chemistry and Physics* **7**, 1925–1945 (2007).
20. Alexander, B. *et al.* Quantifying atmospheric nitrate formation pathways based on a global model of the oxygen isotopic composition (Δ17O) of atmospheric nitrate. *Atmos. Chem. Phys.* **9**, 5043–5056 (2009).
21. Jiang, S. *et al.* Nitrate preservation in snow at Dome A, East Antarctica from ice core concentration and isotope records. *Atmospheric Environment* **213**, 405–412 (2019).
22. Erbland, J., Savarino, J., Morin, S. & Frey, M. M. Nitrate stable isotopes from Antarctic snow: towards new proxies for the interpretation of ice cores. in (2010).
23. Farman, J. C., Gardiner, B. G. & Shanklin, J. D. Large losses of total ozone in Antarctica reveal seasonal ClOx/NOx interaction. *Nature* **315**, 207–210 (1985).
24. Landais, A. *et al.* Firn-air δ15N in modern polar sites and glacial–interglacial ice: a model-data mismatch during glacial periods in Antarctica? *Quaternary Science Reviews* **25**, 49–62 (2006).
25. Kawamura, K. *et al.* Convective mixing of air in firn at four polar sites. *Earth and Planetary Science Letters* **244**, 672–682 (2006).
26. Severinghaus, J. P. *et al.* Deep air convection in the firn at a zero-accumulation site, central Antarctica. *Earth and Planetary Science Letters* **293**, 359–367 (2010).
27. Sofen, E. D. *et al.* WAIS Divide ice core suggests sustained changes in the atmospheric formation pathways of sulfate and nitrate since the 19th century in the extratropical Southern Hemisphere. *Atmospheric Chemistry and Physics* **14**, 5749–5769 (2014).
28. Jiang, S. *et al.* A detailed 2840 year record of explosive volcanism in a shallow ice core from Dome A, East Antarctica. *Journal of Glaciology* **58**, 65–75 (2012).
29. Fegyveresi, J. M. *et al.* Late-Holocene climate evolution at the WAIS Divide site, West Antarctica: bubble number-density estimates. *Journal of Glaciology* **57**, 629–638 (2011).
30. Ding, M. *et al.* Re-assessment of recent (2008 - 2013) surface mass balance over Dome Argus, Antarctica. *POLAR* **35**, (2016).
