# Supplementary material for: Sunlight-driven nitrate loss records Antarctic surface mass balance
Source: Nat Commun. 2022 Jul 25;13:4274. doi: 10.1038/s41467-022-31855-7 (PMC9314437; doi:10.1038/s41467-022-31855-7)
Supplement: Supplementary file 2 — Peer Review File [file 41467_2022_31855_MOESM2_ESM.pdf]

nature portfolio

Peer Review File

Sunlight-driven nitrate loss records Antarctic surface mass balance

Open Access This file is licensed under a Creative Commons Attribution 4.0 International License, which permits use, sharing, adaptation, distribution and reproduction in any medium or format, as long as you give appropriate credit to the original author(s) and the source, provide a link to the Creative Commons license, and indicate if changes were made. In the cases where the authors are anonymous, such as is the case for the reports of anonymous peer reviewers, author attribution should be to 'Anonymous Referee' followed by a clear attribution to the source work. The images or other third party material in this file are included in the article's Creative Commons license, unless indicated otherwise in a credit line to the material. If material is not included in the article's Creative Commons license and your intended use is not permitted by statutory regulation or exceeds the permitted use, you will need to obtain permission directly from the copyright holder. To view a copy of this license, visit http://creativecommons.org/licenses/by/4.0/.

REVIEWER COMMENTS</B>

Reviewer #1 (Remarks to the Author):

This study aimed to apply the δ15N of nitrate remaining on Antarctic snow to estimate surface mass balance (SMB). They concluded that the history of SMB for 700 years could be reconstructed via the analysis of δ15N of nitrate in an ice-core.

This is a unique contribution of the estimation of SMB, which has been a significant challenge in cryosphere science. In addition, this study shed light on the isotope analysis using δ15N of nitrate in snowpack.

The concept of this study was clearly stated. The methodology was technically sounded. Results were significant. The figures were nice. Thus, it was easy to follow the logic of the manuscript.

The writing style needs to be improved. Most readers of this journal may not be experts in cryosphere science and isotope analysis. Therefore, the manuscript should briefly explain common-sense things as a service for the readers.

Please find my comments below.

Introduction

This study seems to assume that the initial δ15N of nitrate value is stable in the Antarctic snowpack. Please explain why the initial δ15N is stable in the Antarctic, although it is already known that reactions in the stratosphere are a major source of nitrate in the Antarctic inland.

Readers of this journal may not understand the methods and costs to measure SMB. Please mention the SMB measurement method to describe why an alternative proxy is needed. (or move the paragraph in line 256 from discussion to introduction)

Figure 2

The equation in Fig. 2(b) is ln(δ15N + 1)=6.98SMB–1 -0.02, implying that the line in Fig 2(b) is the equation on Fig. 2(b). This equation also implies that the vertical and horizontal axis of Fig. 2(b) is ln(δ15N + 1) and SMB–1, respectively. I guess that the line in Fig. 1(b) is eq. (17) (line 420). This is a bit confusing.

Results: SMBδ15N relationship and spatial applicability

Line 139: <40 kg m-1 a–1?

Line 145: The effect of volatilization may be negligible because the δ15N of nitrate is stable in this reaction.

Results: Aurora Basin North SMB reconstruction

The authors analyzed one ice-core to reconstruct SMB for 700 years. However, there is a lack of discussions of the regional representativeness of the ice-core. Indeed, Gautier et al. (2016) revealed that "Local-scale variability, essentially attributed to snowdrift and surface roughness at Dome C, can lead to a non-exhaustive record of volcanic events when a single core is used as the site reference, with a bulk probability of 30 % of missing volcanic events and close to 65 % uncertainty on one volcanic flux measurement (based on the standard deviation obtained from a five-core comparison)." Although it is hard to obtain multiple ice cores, I suggest discussing the uncertainty of the results regarding the representativeness.

Reference: Gautier et al., Clim, Past, 12, 103–113, 2016.

Discussion: Validating the SMBδ15N proxy reconstruction

The authors compared SMBδ15N to SMBdensity to validate the accuracy of SMBδ15N. However, the authors stated that "Ice density-based reconstructions become less effective and more uncertain with depth due to thinning and deformation of ice layers" (line 48). Therefore, the reason why the SMBdensity was selected as a reference should be explained. Additionally, the SMBδ15N can be calculated for other ice-cores, which the δ15N values provided in previous studies. Thus, the authors can reconstruct the SMB for the other sites. It should be a good application of the proposed method. Additionally, some studies may provide the δ15N values and water isotope values of ice-cores, which is another proxy of snow accumulation. As the authors stated in line 51, the reliability of the water isotope values may be low as the proxy of SMB. The authors can reconstruct the SMB by the two proxies and quantitatively indicate the reliability.

Reviewer #2 (Remarks to the Author):

The authors present a novel SMB proxy based on the photolytic fractionation of nitrogen isotopes in nitrate (δ¹⁵N_{NO3}). They describe how they calibrate this proxy using ground based SMB measurements as well as model (MAR) SMB output. They apply this proxy on an ice core retrieved in East Antarctica (Aurora Basin North) and show that it reproduces well the SMB record, when

compared to the SMB record derived from the ice core density and ages. The advantage of this technique is that, with the existing records of nitrogen isotopes (δ¹⁵N_{NO3}) complemented by 92 new measurements, it provides 114 measurements of SMB over the Antarctic continent, when currently “standard” ice core-derived SMB measurements are much more limited.

The results presented in this study are very interesting, the authors describe in a lot of detail how this new proxy is developed, calibrated and provide very useful new δ¹⁵N_{NO3} data measurements, and present these 114 independent SMB measurements over the whole continent. The method described and the measurements provided in this study make this paper highly relevant to the Nature Communications journal and I recommend this manuscript highly for publishing. We just list a number of minor changes that would help make the manuscript clearer to readers. General comments are listed below, while specific comments are provided as pdf changes (see separate document).

General comments

• The authors use a large number of acronyms throughout the manuscript, which in some cases is useful (e.g. SMB_{GPR} which in this instance is the SMB signal due only to upstream surface topography). But perhaps some are unnecessary? For example, why have an explicit distinction between δ¹⁵N_{NO3arc} and δ¹⁵N_{NO3} ? Wouldn’t it be easier to just say the “δ¹⁵N_{NO3} archived”?

• It is never mentioned anywhere whether the SMB values are in ice equivalent, water equivalent or ... ?

• This is more of a syntax comment but I am not sure that using “SMB” in its plural form is necessary. Generally “SMB” suffices, or in some cases, using “SMB values” or “SMB measurements” is better suited

• Perhaps I missed it, but I didn’t see any discussion about the vertical (and therefore temporal) resolution that can be achieved with δ¹⁵N_{NO3} ? It would be interesting to state this explicitly, as well as provide the related temporal resolution of the SMB signal derived. I’m also wondering if the authors could add a (short) discussion about the magnitude of the uncertainties in their calibration in the main manuscript. I understand that the supplement shows the range in the possible regressions but it is not easy to quickly conclude on the size of the errors on the SMB calculated. This would a be great addition to the results and discussion of the manuscript.

Minor comments: see attached document.

Marie Cavitte

Reviewer #3 (Remarks to the Author):

This work presents a new approach for reconstructing surface mass balance on the Antarctic ice sheet based upon the use of the nitrogen isotopes of nitrate (d15N) in snow. This is a clever use of the d15N since nitrate can be lost from snow due to photolysis, and this process is dependent upon snowfall rates (accumulation) and burial out of the photic zone. The manuscript argues that use of this new approach is particularly compelling for the transition zones (ie most of the continent) between the coasts and dry dome summits, where there is more uncertainty with traditional approaches. This work is original and interesting, and enough detail appears in the manuscript and supplement to regenerate the results. The use of the sensitivity studies is very good. In terms of significance, the primary significance here will be to the ice core and glaciology communities and may not be of more general interest.

Overall, the work is very well written but depends on a technical approach, all of which ends up buried in the methods and supplement. In the end, this work feels like a proof-of-concept paper rather than a major step forward in the field. This new approach is certainly compelling and could in the future enhance our understanding more broadly on surface mass balance in Antarctica, but the current form of this manuscript is not convincing that this strategy is more accurate or gives us information we otherwise would not have. While the overall similarity in patterns in Figure 3b are used to support the use of the d15Narc proxy, there are many times when the ice density reconstruction and d15N reconstruction go in opposite directions. And yet, the agreement in “mean value, range, and variability strongly validates” the approach in the work and is used as the major evidence for supporting the conclusion that the d15Narc approach is “better” bc d15N could be measured in lots of places. The only changes of importance that are highlighted in Figure 3c are linked to known climate change events based on other proxies – they do not reveal anything new. And of course there are still very large uncertainties in the final reconstruction based on d15Narc.

The final reconstruction is based on a number of key assumptions that are buried in the supplement. The work would be much improved in a longer format where these key assumptions, and key future data needs, could be more adequately addressed, discussed and promoted to the community for advancement.

Here are a few examples of issues that appear in the supplement:

-maximum values for d15N-nitrate yield unrealistic solutions;

-there’s a clear need for more accurate modeling of e-folding depths and more in situ ground-truthing of remotely determined e-z values and this adds to the uncertainty;

-Suppl Text 1 and Table S1 – why not approach this by changing multiple parameters at once to optimize the fit? This seems like another example where things are tuned rather than allowed to objectively meet

the criteria. (I am not suggesting change all parameters at one, but one by one may be falsifying solutions that together could exist);

-the assumption is made that “non-SMB parameters are not changing over the period of time covered by the SMB reconstruction.” While certain aspects of this can be “forgiven” given the results from the sensitivity calculations it clearly needs to be more forefront in the manuscript that d15N-NO3- must not vary far from the -20 to +20 range and is assumed to be stable over millennia yet large swings are observed in d15N-NO3- in Greenland ice at high accumulation sites, particularly over glacial cycles;

-below the photic zone “the d15N-NO3- is preserved indefinitely” except at very dry sites. Shi et al’s work, which is cited here, shows that earlier assumptions about what the archived d15N values “should be” based on values at the bottom of the photic zone did not in fact match with deeper snowpits across an East Antarctic traverse of snowpits. And the supplement here discusses multiple cores at Dome C and Dome A that differ by nearly 100 per mil in d15N from core to core. It is then suggested that this does not apply at ABN or other sites outside of the driest sites, but where is the evidence for this? How many other places than Dome A and Dome C do we have multiple cores with overlapping depth ranges where we can prove this?

-the idea that NO3- can be redistributed within the ice via “wind pumping and other convective drivers” is a critical issue – how can this be assumed on the one hand to help explain the disagreement with sub-photic zone values but on the other not invalidate the final d15Narc reconstruction?

-the d15N observations end up corrected before being used to account for the different types/depths of snow/firn that were collected via different studies (this is discussed in the main text methods and in the supplement);

-the final reconstructed d15N is compared with observed values and "match" but the ranges of values are huge so the agreement based on color gives a higher sense of certainty than is warranted;

-how does the SMB results compare with other models that depend on density driven changes? how do the results here compare with the GEOS-chem modeling studies that predict d15N across the whole ice sheet as well?

While it is possible to argue against some of the above points as not adding too much additional uncertainty, taken together, this exercise is more of a tuning of a parameter than it is revealing a new proxy by which we can truly advance our science. This work is better suited for a more disciplinary journal, where the details can all be included together. It likely is setting the stage for future important outcomes, but is too nascent at present with the laundry list of assumptions and tuning of parameters (many of which also have very large uncertainties associated with them, even when observed directly).

Responses

Reviewer #1:

The writing style needs to be improved.

We have re-read the manuscript and made changes to make the paper more broadly understandable throughout.

Please explain why the initial δ15N is stable in the Antarctic, although it is already known that reactions in the stratosphere are a major source of nitrate in the Antarctic inland.

Long term observations at Dome C have shown that while there is a clear seasonal cycle in $\delta^{15}N_{NO3}$, year to year averages vary much less. There is a spatial pattern in skin layer $\delta^{15}N_{NO3}$ but these values covary with SMB and are folded into the parameterized Eq 1 as described in Supplemental Text 1.

Readers of this journal may not understand the methods and costs to measure SMB. Please mention the SMB measurement method to describe why an alternative proxy is needed. (or move the paragraph in line 256 from discussion to introduction)

Paragraph in line 256 has been partially moved to introduction. SMB difficulties with existing methods have been made clearer in the introduction.

The equation in Fig. 2(b) is ln(δ15N + 1)=6.98SMB–1 -0.02, implying that the line in Fig 2(b) is the equation on Fig. 2(b). This equation also implies that the vertical and horizontal axis of Fig. 2(b) is ln(δ15N + 1) and SMB–1, respectively. I guess that the line in Fig. 1(b) is eq. (17) (line 420). This is a bit confusing.

In the original draft, we converted the labels on the axis ticks to SMB and $\delta^{15}N_{NO3}$ values because these are more understandable and are the actual variables of interested. We noted this in the caption: *Scatter plot and linear regression of Eq. (1) using all sites in the field dataset, with axis value labeling converted to show simpler SMB and* $\delta^{15}N_{NO3arc}$ *values*. However, to make things clearer, we have added another plot that shows the non-transformed relationship between SMB and $\delta^{15}N_{NO3}$, and we have changed the axes of the original scatter plot to directly reference the values of the regression.

Line 139: <40 kg m-1 a–1?

Corrected.

Line 145: The effect of volatilization may be negligible because the δ15N of nitrate is stable in this reaction.

Mention of volatilization here has been removed for clarity. The negligible isotopic effects of volatilization are still mentioned earlier in the manuscript.

Although it is hard to obtain multiple ice cores, I suggest discussing the uncertainty of the results regarding the representativeness.

We have added an additional paragraph added to discuss the applicability and interpretation of a single ice core in further detail.

However, the authors stated that "Ice density-based reconstructions become less effective and more uncertain with depth due to thinning and deformation of ice layers" (line 48).

Therefore, the reason why the SMBdensity was selected as a reference should be explained.
We have added an additional line added to explain that $SMB_{density}$ has the stated drawbacks and a lower potential resolution, but that it is an established and simple SMB proxy, which makes it good for verification of new proxies.

Additionally, the SMBδ15N can be calculated for other ice-cores, which the δ15N values provided in previous studies. Thus, the authors can reconstruct the SMB for the other sites. It should be a good application of the proposed method. Additionally, some studies may provide the δ15N values and water isotope values of ice-cores, which is another proxy of snow accumulation. As the authors stated in line 51, the reliability of the water isotope values may be low as the proxy of SMB. The authors can reconstruct the SMB by the two proxies and quantitatively indicate the reliability.
To our knowledge, only two other Antarctic ice cores have published $\delta^{15}N$ data: one from WAIS and one from Dome A. Both of these ice cores are outside the geographic bounds that we defined for our proxy, with WAIS both being in West Antarctica and being too wet (i.e., the $\delta^{15}N$ values are barely elevated above atmospheric NO_3^- values), and the Dome A core suffers from the ultra-dry site issues we discuss in the paper. However, we have now added a supplement briefly discussing the results when we apply our proxy to them.

Reviewer #2
The authors use a large number of acronyms throughout the manuscript, which in some cases is useful (e.g. SMB_{GPR} which in this instance is the SMB signal due only to upstream surface topography). But perhaps some are unnecessary? For example, why have an explicit distinction between $\delta^{15}N_{NO3arc}$ and $\delta^{15}N_{NO3}$? Wouldn't it be easier to just say the "$\delta^{15}N_{NO3}$ archived"?
We decided to explicitly call out $\delta^{15}N_{NO3arc}$ compared to $\delta^{15}N_{NO3}$ to be clear that we are only discussing NO_3^- that is in the archived zone and NOT NO_3^- on the surface or in the photic zone. For us, having a simple addition of three letters to the acronym is a good compromise to ensure that we are always being clear and reminding the reader that it is archived NO_3^- without having to constantly fully write out "archived". However, we have also read back though the paper and tried to make sure that no acronyms are added unnecessarily or overused.

It is never mentioned anywhere whether the SMB values are in ice equivalent, water equivalent or ... ?
The SMB values given are listed as kg m^{-2} a^{-1}, which is not dependent upon a depth unit and therefore does not have ice or water equivalent qualifiers. However, we have added a note clarifying that kg m^{-2} a^{-1} is equal to mm-w.e. a^{-1} .

This is more of a syntax comment but I am not sure that using "SMB" in its plural form is necessary. Generally "SMB" suffices, or in some cases, using "SMB values" or "SMB measurements" is better suited
Changed throughout to reflect this.

Perhaps I missed it, but I didn't see any discussion about the vertical (and therefore temporal) resolution that can be achieved with $\delta^{15}N_{NO3}$? It would be interesting to state this explicitly, as well as provide the related temporal resolution of the SMB signal derived.
Paragraph added to address this.

I'm also wondering if the authors could add a (short) discussion about the magnitude of the uncertainties in their calibration in the main manuscript. I understand that the supplement shows the range in the possible regressions but it is not easy to quickly conclude on the size of the errors on the SMB calculated. This would a be great addition to the results and discussion of the manuscript.
We have added this requested information.

Minor Comments
Corrected as suggested. Comments needed responses addressed here:

I think this introduction lacks to state WHY understanding SMB between the coast and the domes is important.
Added more clarification on this point.

I'm wondering why it is not eq 17 of the Methods section that is provided here. Especially since panel b of Fig 2 is giving delta 15Nas a function of SMB and not ln(delta15N+1)
There was some confusion by multiple reviewers in Fig 2 based on our labeling of the axes with converted values (which we thought would be more useful to readers than having log(δ^{15}N+1) and SMB^{-1} values). To avoid confusion, we have reverted to having the non-converted values on the figure axes and also added a plot showing the relationship between δ^{15}NNO3 and SMB without the linear transformation.

shouldn't this SMB also be called SMBdelta15N
Here in this equation, we are simply expressing that SMB (the real world SMB) is related to δ^{15}N in a linear fashion. We reserve the term $SMB_{\delta 15N}$ specifically for SMB values that are reconstructed from δ^{15}N (and that are modeled estimates of the real world SMB).

(1) This figure is difficult to read because it is too small. It's difficult to read the name of the places on the map, as well as the legend in panel a. On panel b as well, the legend is too small and the symbols are too small. I would recomment putting the two panels on top of each other so that their widths can be increased.
This figure has been changed now closer to this suggestion.
(2)
it would be nice to mark a difference between the previously measured sites, and the data newly reported here.
We tried several ways to distinguish these samples as you suggested. Unfortunately, we could not find a symbology that clearly showed this information without taking away from the other important information or making the figure more confusing.

Is that the right reference? (for Fig 3b caption)

Yes, the Servettaz thesis contained his reconstruction of GPR based accumulation for the upstream transect.

I find it a little unusual to describe this way of calculating SMB as a SMBdensity measurement. This is quite a standard way of measuring SMB from ice cores. So I would intuitively just call this "SMBice core". And here I would say "SMB calculated using the physical density of the core and the depth-age curve from the core".
Edited to phrase the description more as you state. However, while the SMB calculated from density is a standard ice core technique, we think it still should be identified distinctly as $SMB_{density}$ to 1) explicitly remind the reader what this particular SMB reconstruction is derived from, 2) stay consistent in terminology for all SMB methods used in the manuscript, and 3) avoid giving the impression that the density based SMB record is 100% accurate and representative of the historical SMB (as being simply named SMBicecore would suggest), since there are also problems that exist for $SMB_{density}$ related to incomplete/damaged/broken core segments.

Why use 1-m sections? And not the annual resolution as is probably provided by the ABN core? Is it because it is closer to the temporal resolution of the SMBdelta15N?
Added a sentence clarifying that the $SMB_{density}$ record is limited to 1m resolution because that is the resolution of the density measurements themselves, since density is measured on each 1 m core segment. An annually resolved density record created using the age-depth model simply be an interpolation assuming constant density for each segment, which we know is not true, particularly for the uppermost core. As a results, an “annual” density record would be misleading as it suggests a resolution of measured density variability that we did not actually have.

Reference 41 shows radar data between Dome C and Vostok. There is no radar data that links to the ABN basin. So I am not sure that this reference is the right one ?
Reference 41 (now 49) is a paper that describes the methodology also later used for ABN. That data itself does not have its own published paper, but is referenced by the Servettaz thesis. The reference 41 has been moved to immediately after the GPR to distinguish that it is referencing the technique and not the ABN data.

I don't understand what is meant here, could it be reworded? It seems that it is packing a lot of concepts into one.
Rephrased for simplicity: These surface features can be identified as buried horizons to depths below the deepest segment of ABN1314-103, which suggests that they have been stable features of the local landscape for hundreds of years.

This is too vague. Could the authors describe exactly how this is done ?
This section has been significantly revised for clarity.

Perhaps I missed it but what is the vertical resolution that can be achieved with these delta15N measurements and therefore the tempral resolution of the SMB record that can be obtained?
Paragraph added to Results: $SMB_{\delta 15N}$ relationship and spatial applicability about resolution potential.

Perhaps I didn't follow but why 3ze ? Is it just because it marks the limit with the photic zone?
Z_e is the depth where there is 1/e of the initial irradiance present. 3x this depth ($3z_e$) is where 95% of the irradiance is gone and is typically taken as the bottom of the photic zone.

why calculate a single value? This is not explained
Each single sampling location (i.e., a single pit or a single core) is represented by a single $\delta^{15}N$ value representing the mean value for the site. This is to 1) reduce the impact of short term SMB variability because our SMB site values are based on longer term averages and to 2) give each sample the same weighting of 1 in the final regression (rather than having a single core with 50+ values comprise a large percent of the overall field sample database).

higher and more interior than what?
Reworded to state that it overestimates at "high elevation (>3000 m) interior sites"

I think it could be worth mentioning how these two overlapping subsets of sites were chosen.
Clarified to state that they were split to be near equal size near a naturally occurring break.

Reviewer #3
We appreciate the comments and suggestions made by the reviewer and have attempted to address them to our best ability. Much of their criticisms are rooted in details allegedly "buried" in the methods and supplement, but we disagree with this characterization. Due to the limited word space for the main text and a need for a broad communication of the overall research, we relegated the more technical supporting details (with interest to a small subset of the readers) to the supplement. We feel that we were always very clear in stating known drawbacks and uncertainties in the main text and directing the reader to the supplement for more information. However, we have revised the main text in an effort to be even more explicit linking the supplemental information to the main text, and to be very upfront about the unknowns and drawbacks of our study.

However, at times it appears that this reviewer had a mental concept of what they wished our paper was (e.g., deep chemical reaction/transport modeling, a thorough review that compiles a comprehensive understanding of all nitrate dynamics across Antarctica, precise values for parameters that do not currently exist) and critiqued our manuscript relative to this concept rather than reviewing our work for what it actually was, intended to be, and accomplished. In particular, much of the review was treating the sensitivity testing section of the supplemental as the primary focus of the paper, instead of it serving the supplemental role it was designed to be. While this would be a (different) useful paper, it's highly specialized nature would be more appropriate for a very technical journal, and detract from the current manuscript's broad focus for a general audience. It also seems at times that we are unfairly being punished in this review for being open and inclusive about all of the assumptions and uncertainties involved in our study. For example, we chose to include a description of all necessary actions we made to standardize our field data across different

sampling methods and studies, but the reviewer included this as a problematic "issue" of our research.

We are not dismissing this review (the reviewer made several good comments despite an overall dismissive conclusion), and we have made changes or clarifications in light of many of their points. However, when considering the critique from Reviewer 3, we hope that the editor takes into account our other two reviews, and particularly the counterpoint from reviewer 2 who gave a very thorough review that highly recommended our work as deserving to be published in Nature Communications.

Specific responses to reviewer comments:
-maximum values for d15N-nitrate yield unrealistic solutions;
We are not exactly sure what is meant by this comment. Perhaps one of these answers will address what was intended:
1) The maximum values observed in Antarctic for $\delta^{15}N_{NO3}$ are generally 300–400 ‰, which corresponds to SMB = 20–25 kg m^{-2} a^{-1}. This matches very well with the minimum SMB observed across Antarctica (generally, 19-30 kg m^{-2} a^{-1} at Vostok and Dome Summit).
2) The unrealistic $\delta^{15}N$ values predicted at sites like the blue ice regions is because the accumulation is practically non-existent or negative, which violates the basic theoretical concept behind our proxy (which requires NO_3^- to be archived over time through snow burial), and this is stated in the manuscript.
3) The very high slope of the maximum regression in the sensitivity testing should be unrealistically high, because it uses the maximum estimates from the value range for each parameter in the model, and they are all multiplied against each other in the model's equation.
-there's a clear need for more accurate modeling of e-folding depths and more in situ ground-truthing of remotely determined e-z values and this adds to the uncertainty;
Yes, we freely acknowledged this as a critical future need and a likely major source of the imprecision in our regression. However, the lack of e-folding depth data and models is information lacking for the entire Antarctic cryosphere discipline, and is not simply an oversight on our parts.
-Suppl Text 1 and Table S1 – why not approach this by changing multiple parameters at once to optimize the fit? This seems like another example where things are tuned rather than allowed to objectively meet the criteria. (I am not suggesting change all parameters at one, but one by one may be falsifying solutions that together could exist);
The intent of the sensitivity tests was not to determine which exact set of parameter values is the most likely "true value combination", but rather if 1) we could reproduce the field observed relationship with parameter values that were realistic and 2) how much potential variability could be observed in $\delta^{15}N_{NO3arc}$ values for parameters that we treated as constant (i.e., how much "noise" were we accepting in our regression from parameter variability in exchange for simpler modeling).

As we noted in the supplemental text, there are many different solutions that match the observed field relationship using different parameter combinations, including changing only the quantum yield and otherwise using the best estimates for other parameters. Without better constraints on the quantum yield, it would seem that almost any combination of the

other parameters could be made to work by using a quantum yield value that falls well within the range of published values. Thus, we didn't attempt to define a specific "best parameter estimates", but used the sensitivity testing to answer the two questions listed above.

-the assumption is made that "non-SMB parameters are not changing over the period of time covered by the SMB reconstruction." While certain aspects of this can be "forgiven" given the results from the sensitivity calculations it clearly needs to be more forefront in the manuscript that d15N-NO3- must not vary far from the -20 to +20 range and is assumed to be stable over millennia yet large swings are observed in d15N-NO3- in Greenland ice at high accumulation sites, particularly over glacial cycles;
We did state early in the manuscript that this approach is best restricted to the Holocene because of known changes to the environment during glacial periods that probably alter some of the foundational assumptions in ways we have not yet been able to quantify. We have now added additional clarification regarding this point, including a reference to a paper that showed how the large $\delta^{15}N_{NO3}$ changes in Greenland could be at least partly explained through SMB changes.

-below the photic zone "the d15N-NO3- is preserved indefinitely" except at very dry sites. Shi et al's work, which is cited here, shows that earlier assumptions about what the archived d15N values "should be" based on values at the bottom of the photic zone did not in fact match with deeper snowpits across an East Antarctic traverse of snowpits. And the supplement here discusses multiple cores at Dome C and Dome A that differ by nearly 100 per mil in d15N from core to core. It is then suggested that this does not apply at ABN or other sites outside of the driest sites, but where is the evidence for this? How many other places than Dome A and Dome C do we have multiple cores with overlapping depth ranges where we can prove this?
Shi et al. 2015's criticism of predicting what $\delta^{15}N_{NO3arc}$ values should be was primarily focused on Erbland 2013's asymptotic approach that tried to extrapolate $\delta^{15}N_{NO3arc}$ values from pits as shallow as 20 cm. Shi showed that this approach works better if you have deeper pits and more samples. However, we did not use extrapolated $\delta^{15}N_{NO3arc}$ values in our field samples but rather calculated a mean $\delta^{15}N$ value from the individual pit sample values. The most severe changes with depth were generally observed by Shi et al. either at the driest sites (which we discuss in detail in the supplement) or more in the $\delta^{18}O$ (which we are not analyzing here).

Our supplement does not discuss how different cores differ by 100 ‰ in $\delta^{15}N_{NO3}$ at Dome A and Dome C, but rather that the deeper core values are nearly 100 ‰ lower than near surface $\delta^{15}N_{NO3}$ values. Thus, this isn't a problem of parallel core replicability, but rather that additional post-depositional changes are occurring at depths where NO_3^- was thought to be stable. This is what we discuss in the supplement, and we explain the reasons why this effect is exaggerated and severe at the driest sites : "This effect would be enhanced at the driest sites because firn convection zones are deeper at drier sites[25,26], the very low NO_3^- concentrations (~10-50 ng g^{-1}) in the archived zone at drier sites are sensitive to even small additions of re-oxidized NO_3^-, and slow burial rates keep NO_3^- longer in the zone where interstitial firn air can exchange."

-the idea that NO3- can be redistributed within the ice via "wind pumping and other convective drivers" is a critical issue – how can this be assumed on the one hand to help explain the disagreement with sub-photic zone values but on the other not invalidate the final d15Narc reconstruction?
As we discuss in the supplement and note in the main text, this effect is exaggerated at the driest sites (which is why we do not recommend the proxy for exact quantitative reconstructions at sites where SMB < 40 kg m^{-2} a^{-1}) due to the known increase in deep ventilation at dry sites and the extremely low NO_3^- concentrations that are thus sensitive to even small redistributions of NO_3^- from shallower snow. While there is admittedly not a large number of deep pits or cores with $\delta^{15}N$ data at the intermediate or wetter zones, those that do exist do not show the same profile changes as we see at the ultra-dry sites. And while this is a bit of circular reasoning, we would expect the ABN $SMB_{\delta15N}$ reconstruction to be offset significantly from the $SMB_{density}$ values if there was a similar degree of wind pumped NO_3^- delivery at ABN, as $SMB_{\delta15N}$ values reconstructed for the Dome C deep pits and core $\delta^{15}N$ values are 40-50 kg m^{-2} a^{-1}, compared to actual SMB of 25 kg m^{-2} a^{-1} at the site.

-the d15N observations end up corrected before being used to account for the different types/depths of snow/firn that were collected via different studies (this is discussed in the main text methods and in the supplement);
We don't find this to be a problem, as we explain that it is only Noro et al's data that was corrected, and this was necessary because they only took a single value from the entire mixed surface to bottom pit profile. Our regression does not significantly change if you remove Noro's data (and in fact slightly improves), but we included the data to be more spatially representative. Our detailed discussion of how we corrected the data was included here to be fully forthright about our analysis.
-the final reconstructed d15N is compared with observed values and "match" but the ranges of values are huge so the agreement based on color gives a higher sense of certainty than is warranted;
We are not sure of a better way to illustrate this data on the map figure other than a simple color gradient across the full range of data. If a more exact comparison is desired, the scatter plot with regression clearly shows the spread and nature of residuals from the regression trend.
-how does the SMB results compare with other models that depend on density driven changes? how do the results here compare with the GEOS-chem modeling studies that predict d15N across the whole ice sheet as well?
We assume the reference here is to Zatko et al. 2016's modeling. We have added an additional citation and note directly linking to this study into the main text where we discuss the modeled spatial distribution of $\delta^{15}N_{NO3}$. However, we are unable to find the data from Zatko 2016 available outside of their small figure 11c. We can make some broad comparisons, but without the actual data itself in the form of a geotiff or other GIS-friendly form, it is hard to make any actual comparative analyses.

REVIEWERS' COMMENTS

Reviewer #1 (Remarks to the Author):

This study aimed to apply the δ15N of nitrate remaining on Antarctic snow to estimate surface mass balance (SMB). They concluded that the history of SMB for 700 years could be reconstructed via the analysis of δ15N of nitrate in an ice-core.
I have already reviewed this manuscript. In response to my review comments, the authors have made appropriate revisions to the manuscript. Therefore, I have no additional comments. I evaluated the manuscript as worthy of publication in this journal.

Reviewer #2 (Remarks to the Author):

The authors have responded to all the points mentioned and so we recommend this manuscript for publication.
We have no further points to add.

Reviewer #3 (Remarks to the Author):

The manuscript is improved based upon the response to reviewers. While the authors clearly feel sensitive about questioning the large uncertainties and underlying assumptions associated with their proposed technique, the questioning was not unwarranted. The other reviewers clearly indicated a need for several clarifications, and while the other reviewers were more excited about a potential replacement technique for SMB density based estimation, it was necessary for the authors to be more clear (by adding text) about why this technique (with its caveats) is still an important advancement in the field. The level of technical understanding required would be better reviewed in a disciplinary journal -- the authors argue that the "limited word space and a need for broad communication of the overall research" in Nature Comm requires them to focus on the broadest possible application of their research. This in fact argues for a more technical space to administer the findings with a future broader application more appropriate for Nature Comm. i.e. the authors are "choosing" this broad approach.

The work is much improved with the clarifying text and additional calculations/applications added in with the revision. The work is noteworthy and the methodology is sound as it stands now. There is limited scope under which this technique is likely to apply until proven otherwise (i.e more studies, more sampling along with ground-truthing SMB is required to substantiate the utility of application in different environments).

A few minor comments:
-line 89, change this to "should be a very minor component" rather than "is". other works that include co-authors on this paper have argued in other publications that there are no "field observations" of volatilization.
-line 113, "either" does not make sense here

-Figure 2 is improved from last version but it is necessary to have the high-resolution image to read it
-several times in the manuscript it is mentioned what an important logistical improvement this method allows for, because (for instance) relatively small sample sizes are required. 100 nmol of NO3- is not necessary for all labs -- there are 2 labs in China, 1 in Japan and at least 4 off the top of my head in the US (Stanford, Princeton, Univ of Washington, Brown Univ) who run samples with the denitrifier method at sample sizes much lower than 100 nmol. There are also labs that do not require use of the resin concentrating method and are still able to resolve low concentration samples. The manuscript should not state the need for 100 nmol as a requirement that can be improved - it already is improved upon currently! (line 193, 382, 513-514 - also the references 21 and 23 don't make sense here?)
-Figure 4 c has a typo in the subheading on the figure
-Figure 5: i'm not sure this figure is necessary in the main text

Responses to Reviewer #3:

-line 89, change this to "should be a very minor component" rather than "is". other works that include co-authors on this paper have argued in other publications that there are no "field observations" of volatilization.
Changed

-line 113, "either" does not make sense here
Edited to make sense

-Figure 2 is improved from last version but it is necessary to have the high-resolution image to read it
Smallest parts and texts made larger and more legible

-several times in the manuscript it is mentioned what an important logistical improvement this method allows for, because (for instance) relatively small sample sizes are required. 100 nmol of NO3- is not necessary for all labs -- there are 2 labs in China, 1 in Japan and at least 4 off the top of my head in the US (Stanford, Princeton, Univ of Washington, Brown Univ) who run samples with the denitrifier method at sample sizes much lower than 100 nmol. There are also labs that do not require use of the resin concentrating method and are still able to resolve low concentration samples. The manuscript should not state the need for 100 nmol as a requirement that can be improved - it already is improved upon currently! (line 193, 382, 513-514 - also the references 21 and 23 don't make sense here?)
The 100 nmol refers to the minimum value needed if oxygen isotope anomaly analysis is desired along with the nitrogen isotopes. This is standard practice in many labs, but we did not make this clear in the article, and the reviewer is correct that only 5 nmol are needed if solely nitrogen isotopes are needed. We have changed the references to this need throughout to reflect the smaller minimum requirements.

References removed (they were references for the concentration of nitrate in Antarctic snow, which was used to calculate the required snow amounts, but this was not made clear in the text).

-Figure 4 c has a typo in the subheading on the figure
This appears to be an odd artefact of our Word processing, as it is not evident on the actual image file. We have confirmed that our uploaded version is ok.

-Figure 5: i'm not sure this figure is necessary in the main text
We feel that it adds visual context to illustrate the complexity of reconstructing SMB histories, and since we are well below the 10 figure limit for illustrations, we believe that its inclusion does not distract from the main text. If the editor disagrees, we are ok with moving it to the supplementary.
